# Electro‐Mechanochemical Atom Transfer Radical Cyclizations using Piezoelectric BaTiO_3_

**DOI:** 10.1002/anie.202003565

**Published:** 2020-07-09

**Authors:** Christian Schumacher, José G. Hernández, Carsten Bolm

**Affiliations:** ^1^ Institute of Organic Chemistry RWTH Aachen University Landoltweg 1 52074 Aachen Germany

**Keywords:** ATRC reaction, ball milling, BaTiO_3_, mechanochemistry, piezoelectric materials

## Abstract

The formation and regeneration of active Cu^I^ species is a fundamental mechanistic step in copper‐catalyzed atom transfer radical cyclizations (ATRC). Typically, the presence of the catalytically active Cu^I^ species in the reaction mixture is secured by using high Cu^I^ catalyst loadings or the addition of complementary reducing agents. In this study it is demonstrated how the piezoelectric properties of barium titanate (BaTiO_3_) can be harnessed by mechanical ball milling to induce electrical polarization in the strained piezomaterial. This strategy enables the conversion of mechanical energy into electrical energy, leading to the reduction of a Cu^II^ precatalyst into the active Cu^I^ species in copper‐catalyzed mechanochemical solvent‐free ATRC reactions.

## Graphical abstract

**Squeezing electrons by milling**: Macroscopic mechanical forces transduced by ball milling are capable of inducing electrical polarization in piezoelectric BaTiO_3_. The electropotential of the strained piezoelectric material was harvested to trigger the reduction of a Cu^II^ complex into an active Cu^I^ catalyst for mechanochemical solvent‐free ATRC reactions.

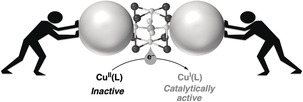

The ability of metal complexes to change their oxidation states is fundamental in catalysis, and achieving a complete control of this process is a long‐sought‐after goal of synthetic organic chemists. Currently, one of the most effective strategies to tune the redox properties of reactants in a chemical reaction is by implementation of electrochemistry through the direct application of an electrical potential in electrochemical cells.^1^ However, other stimuli, such as light or mechanical force, are also known to induce similar redox changes in matter. For example, grinding of the mineral cinnabar (HgS) with vinegar in a copper mortar has been known since antiquity to reduce mercury(II) sulfide into elemental mercury.^2^ Also, as early as 1893 Lea reported a mechanical reduction of hexacyanoferrate (III) ions by manual grinding that led to the mechanochemical formation of Fe^II^ and Fe^III^ species.^3^ In a more recent example, Yan et al., demonstrated that high pressures applied on a copper(I) *m*‐carborane‐9‐thiolate complex induced a redox reaction resulting in the formation of copper (0) concomitantly with the generation of new sulfur–sulfur bonds.^4^

Historically, attempts to merge electric and mechanical activation modes for chemical synthesis have been made, such as the development of electric‐assisted ball milling. This approach relies on the application of low‐current, high‐voltage electrical impulses during ball milling to accelerate chemical transformations and to afford products sometimes inaccessible by standard ball milling techniques.^5^ Although effective, electric‐assisted ball mills require an external power supply and additional connecting devices, rendering the ball milling process complex. However, there is no doubt that the combination of electrochemistry and mechanochemistry could be highly synergistic and may lead to new frontiers in catalysis research. On this basis, we became curious if mechanical forces transduced by ball milling could be directly harnessed to induce electrical polarization in piezoelectric materials.^6^ If achievable, the strained piezoelectric material would develop domains in its structure that could resemble both electrodes found in electrochemical cells thereby enabling chemical reactions influenced by mechanical force and electric fields. Until now, activation of piezoelectric materials for catalysis has been predominantly limited to the use of acoustic cavitation by ultrasonication in solution.^7^ Hence, we set to investigate whether the intrinsic mechanical dynamics operating inside a ball mill could be leveraged to induce piezoelectricity in materials such as barium titanate (BaTiO_3_) (Figure 1 a).^8^

**Figure 1:**
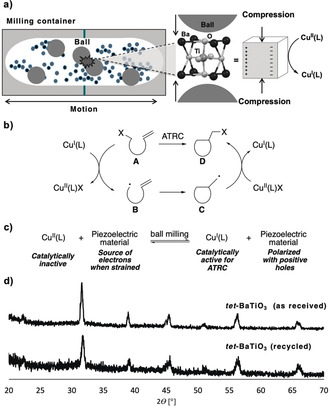
a) Mechanical activation of piezoelectric materials (for example, BaTiO_3_) in a ball mill. b) Postulated mechanism of copper‐catalyzed ATRC reactions. c) Reduction of inactive Cu^II^ complexes into catalytically active Cu^I^ species by piezoelectric materials under ball milling conditions. d) PXRD patterns of piezoelectric BaTiO_3_ samples.

To test this idea, we selected the copper‐catalyzed atom transfer radical cyclization (ATRC) reaction. In ATRCs, copper(I) complexes promote the generation of radicals from alkyl halides **A** through reversible redox processes (Figure 1 b). The formed carbon‐centered radicals **B** undergo intramolecular cyclizations to afford products **C** through the formation of new C−C bonds. Finally, the in situ generated Cu^II^ complex transfers back the halide atom to **C** providing the corresponding ATRC products **D**. This last step promotes the quantitative regeneration of the catalytic Cu^I^ species (Figure 1 b).^9^ However, in ATRC reactions there is often an accumulation of Cu^II^ deactivator species due to radical–radical couplings and disproportionation background reactions.^9^ Moreover, oxidation of the Cu^I^ catalyst by oxygen can render inactive Cu^II^ species, which slow down the ATRC.^10^ Based on these precedents, our rationale was that the lack of catalytic activity by copper(II) complexes in ATRCs was a desirable property to determine if the reduction of a Cu^II^ precatalyst into active Cu^I^ species could be triggered by strained piezoelectric materials during a mechanochemical ATRC by ball milling (Figure 1 c). If required, the electric field obtained from the strained piezoelectric material could also possibly prevent the build‐up of copper(II) parasitic species in the reaction mixture, thus maintaining the progress of the reaction.

As a model system we selected monobromoacetamide **1 a** as the alkyl halide, Cu(OTf)_2_ as the copper salt and solid tris(2‐pyridylmethyl)amine (TPMA) as the ligand for a mechanochemical ATRC. Initial milling experiments of **1 a** in a mixer mill in the absence or in the presence of either copper(II) triflate (5.0 mol %) or TPMA (30 mol %) attested its mechanical stability since **1 a** was recovered unchanged after 90 min of milling at 25 Hz (for details, see the Supporting Information). However, when a mixture of alkyl halide **1 a**, Cu(OTf)_2_ (5.0 mol %) and TPMA (30 mol %) was ground under the same reaction conditions full consumption of **1 a** was detected by ^1^H NMR spectroscopy (Table 1, entry 1). At first, this result was puzzling since the copper complex [Cu^II^(TPMA)(OTf)_2_] expected to form in situ was anticipated to be inactive in the ATRC. However, a series of experiments lowering the amount of TPMA demonstrated that the excess of ligand had simultaneously acted as a reducing agent rendering active Cu^I^ species.^11^ Therefore, to suppress the background formation of **2 a** caused by the excess of TPMA, a catalytic system composed of Cu(OTf)_2_ (5.0 mol %) and TPMA (4.5 mol %) was chosen (Table 1, entry 4). Under these reaction conditions only traces of **2 a** were observed, which was the indispensable requirement to study the ability of piezoelectric materials to trigger an ATRC.

**Table 1:** Effect of the ligand loading on the copper‐catalyzed mechanochemical ATRC reaction of **1 a** in a mixer mill.^[a]^ 
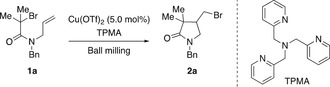

Entry	TPMA [mol %]	**1 a**:**2 a** [%]^[b]^
1	30	1:99
2	15	1:99
3	10	42:58
**4**	**4.5**	**98**:**2**

[a] Reaction conditions: **1 a** (100 mg, 0.34 mmol), Cu(OTf)_2_ (6.1 mg, 0.017 mmol, 5.0 mol %) and ligand were charged inside a 10 mL ZrO_2_ milling jar using one ZrO_2_ ball (10 mm in diameter), under argon atmosphere. The mixture was milled at 25 Hz for 90 min. [b] Determined by ^1^H NMR spectroscopy.

Then, alkyl halide **1 a**, Cu(OTf)_2_ (5.0 mol %) and TPMA (4.5 mol %) were milled in the presence of tetragonal BaTiO_3_ nanoparticles of 200 nm and 500 nm in diameter (Table 2, entries 1 and 2). The analysis of the milled mixture by ^1^H NMR spectroscopy revealed that in both cases the presence of BaTiO_3_ during the milling had clearly favored the formation of product **2 a** (Table 2, entries 1 and 2 vs. Table 1, entry 4).^12^ Encouraged by these results, and recognizing that the changes in electrical polarization of the piezoelectric material would depend on both the number and the strength of the collisions experienced by BaTiO_3_ in the ball mill, the ATRC reaction was repeated using a larger number of balls, while the original total mass of the ball bearing was kept constant (Table 2, entries 3 and 4). These experiments showed that not only the number but also the strength of the collisions had an amplifying effect on the performance of piezoelectric *tet*‐BaTiO_3_. For example, the use of eight milling balls of 5 mm in diameter significantly favored the formation of product **2 a** (Table 2, entries 2 and 3). However, a similar reaction using 39 milling balls of 3 mm in diameter had only a modest improvement in the reaction (Table 2, entries 2 and 4). These results indicated that securing a large number of impacts inside the milling container was not enough prerequisite for the activation of *tet*‐BaTiO_3_. Only individual collisions capable of exerting sufficient mechanical force would activate the piezoelectric material by milling. This idea was corroborated after repeating the same experiment but at a higher milling frequency (30 Hz vs. 25 Hz). Under these new milling conditions, the formation of product **2 a** was greatly improved (Table 2, entries 4 and 5), which is most likely due to the larger number of colliding events of higher linear momentum achieved at 30 Hz. Additionally, ball milling with multiple balls (that is, eight milling balls of 5 mm in diameter) enabled reducing the amount of *tet*‐BaTiO_3_ from 40 wt % to 10 wt % without affecting the yield of the ATRC reaction in the mixer mill (Table 2, entries 6 and 7). Attempts to carry out the ATRC reaction in a planetary ball mill^13^ proved feasible as well, although significantly lower amounts of product **2 a** were obtained (Supporting Information, Tables S3 and S6).

**Table 2:** Copper‐catalyzed mechanochemical ATRC reaction of **1 a** in the presence of piezoelectric and non‐piezoelectric additives.^[a]^ 
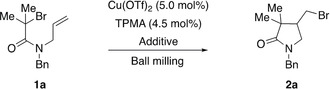

Entry	Additive	Additive [wt %]^[b]^	Number of balls (Ø)^[c]^	**1 a**:**2 a** [%]^[d]^
1	*tet*‐BaTiO_3_ (200 nm)	40	1 (10 mm)	71:29
2	*tet*‐BaTiO_3_ (500 nm)	40	1 (10 mm)	68:32
3	*tet*‐BaTiO_3_ (500 nm)	40	8 (5 mm)	3:97
4	*tet*‐BaTiO_3_ (500 nm)	40	39 (3 mm)	60:40
5	*tet*‐BaTiO_3_ (500 nm)	40	39 (3 mm)	4:96^[e]^
**6**	***tet*** **‐BaTiO_3_ (500 nm)**	**20**	**8 (5** **mm)**	**3**:**97** ^[f]^
7	*tet*‐BaTiO_3_ (500 nm)	10	8 (5 mm)	1:99
8	TiO_2_ (anatase)	20	8 (5 mm)	94:6
9	Al_2_O_3_ (gamma)^[g]^	20	8 (5 mm)	95:5
10	SrTiO_3_	20	8 (5 mm)	50:50
11	ZnO (18 nm)	40	8 (5 mm)	8:92
12	*cub*‐BaTiO_3_ (100 nm)	40	8 (5 mm)	3:97

[a] Reaction conditions: **1 a** (100 mg, 0.34 mmol), Cu(OTf)_2_ (6.1 mg, 0.017 mmol, 5.0 mol %), TPMA (4.4 mg, 0.015 mmol, 4.5 mol %) and the additive were charged inside a 10 mL ZrO_2_ milling jar using ZrO_2_ ball bearing under argon atmosphere and milled at 25 Hz for 90 min. [b] The weight percentage of the additive is calculated in relation to the overall reaction mixture mass (substrate, copper salt, and ligand). [c] 1×10 mm ZrO_2_ ball=3.45 g; 39×3 mm ZrO_2_ ball=3.40 g; 8×5 mm ZrO_2_ ball=3.33 g. [d] Determined by ^1^H NMR spectroscopy, each **1 a**:**2 a** ratio value corresponds to the average of four independent reactions. [e] Milling experiment at 30 Hz. [f] Control experiments by quadruplicate in the absence of *tet*‐BaTiO_3_, copper salt, or TPMA using eight milling balls did not promote the formation of **2 a** in comparable values (see the Supporting Information). [g] Al_2_O_3_ for chromatography, mainly gamma‐type aluminum oxide crystalline form according to the commercial supplier (see the Supporting Information).

At this point of the research we had demonstrated that the presence of *tet*‐BaTiO_3_ was indispensable for the ball milling ATRC reaction. As illustrated in Figure 1 c, mechano‐induced electron transfer from the piezoelectric *tet*‐BaTiO_3_ is suggested to generate catalytically active Cu^I^ species from [Cu^II^(TPMA)(OTf)_2_]. To maintain electron neutrality in the system, *tet*‐BaTiO_3_ is expected to develop positive electron holes (Figure 1 c), which could have been stabilized by the presence of the remaining counterion (not shown in Figure 1 c). Moreover, even though TPMA was used as limiting reagent in the in situ formation of [Cu^II^(TPMA)(OTf)_2_], it is plausible that TPMA could have served as the sacrificial electron donor in this system (oxidation potential of TPMA=+1.04 V vs. SCE).7d Since only traces of Cu^I^ might have been necessary to initiate the ATRC catalytic cycle.

Notably, control experiments done in quadruplicate using non‐piezoelectric solid additives such as TiO_2_ and Al_2_O_3_ only afforded trace amounts of product **2 a** (Table 2, entries 8 and 9). Moreover, UV/Vis analysis of milled mixtures of Cu(OTf)_2_ and TPMA in the presence of *tet*‐BaTiO_3_ or Al_2_O_3_ revealed spectral differences in the samples in terms of wavelength of maximum absorption and absorbance, suggesting that Cu^II^ species underwent redox processes when piezoelectric *tet*‐BaTiO_3_ was present in the milling experiment (Supporting Information, Figure S2). Additionally, the use of SrTiO_3_, a material that has been reported to exhibit ferroelectricity at room temperature when strained, led to moderate reactivity (Table 2, entry 10).^14^ On the other hand, when piezoelectric zinc oxide was used as an additive,7d, 7g the mechanochemical ATRC was significantly reactivated (Table 2, entry 11). Similarly, cubic barium titanate nanoparticles proved active to favor the ATRC by ball milling (Table 2, entries 12). At first, this result was surprising because pristine *cub*‐BaTiO_3_ is highly symmetric and it would develop a weaker macroscopic polarization upon strain when compared to *tet*‐BaTiO_3_.^8^, ^15^ However, *cub*‐BaTiO_3_ has also proven piezoelectric active in mechanochemical atom transfer radical polymerizations by ultrasonication in solution.7c Moreover, while this work was reviewed, Ito and co‐workers reported the ability of *cub*‐BaTiO_3_ to reduce aryl diazonium salts upon ball milling.^16^ Importantly, the ability of piezoelectric materials to effectively trigger reaction by mechanochemistry is expected to depend not only on the polarizability of the material (that is, their dielectric constants) or its crystal structure. Other parameters such as the particle size of the piezomaterial should also be considered, since mechano‐induced electron transfer events between piezoelectric materials and reactants by ball milling are anticipated to occur at their interface.

Having identified the best milling conditions to use the piezoelectric properties of *tet*‐BaTiO_3_ for mechanochemical ATRC reactions, we selected a few additional representative examples to further test this concept. Owing to the readily availability of BaTiO_3_, its recyclability after the reaction (Figure 1 d),^17^ and the stochastic nature of the activation of BaTiO_3_ by ball milling, 20 wt % of *tet*‐BaTiO_3_ (500 nm) was selected as the best loading. Subjecting *N*‐Me‐monobromoacetamide **1 b** to the standard reaction conditions gave ATRC product **2 b** in 73 % yield after 90 min of milling (Scheme 1). The use of tertiary acetamides was found essential for the success of the reaction, since substrates such as *N*‐H free acetamide **1 a**‐*N*H failed at undergoing the ATRC reaction. This may be the result of competition between free acetamide and TPMA ligand for the copper metal center. On the other hand, *N*‐benzyl‐*N*‐(2‐methylallyl) acetamide (**1 c**) and *N*‐benzyl‐*N*‐(but‐2‐en‐1‐yl)acetamide (*E*:*Z* 83:17; **1 d**) reacted smoothly to give cyclized products **2 c** and **2 d** in 71 % yield and 85 % yield, respectively (Scheme 1).

**Scheme 1:**
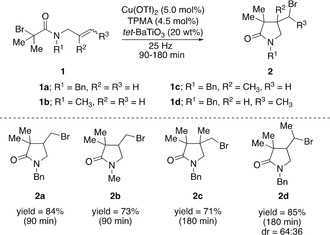
Mechanochemical ATRC examples using *tet*‐BaTiO_3_. Yields after column chromatography.

In summary, we have demonstrated how mechanical activation of piezoelectric materials by ball milling enables precise control over the oxidation state of ligand‐stabilized metal complexes, and its application in mechanically induced copper‐catalyzed atom transfer radical cyclizations. Mechanical stimulation of piezoelectric tetragonal BaTiO_3_ nanoparticles was found to depend on both the number and the strength of colliding events experienced by BaTiO_3_ inside the milling vessel. Systematic fine‐tuning of the milling parameters led to the identification of the best ATRC conditions to convert monobromoacetamides **1 a–d** into the corresponding lactams **2 a**–**d** in good yields. The results of this proof‐of‐concept study demonstrate the feasibility to productively merge electro‐ and mechanical activation modes in mechanochemical reactions by ball milling, and will certainly guide future studies on the use of piezoelectric materials not only as additives^16^ but also, as in the present work, in a catalytic fashion.

## Conflict of interest

The authors declare no conflict of interest.

## Supporting information

As a service to our authors and readers, this journal provides supporting information supplied by the authors. Such materials are peer reviewed and may be re‐organized for online delivery, but are not copy‐edited or typeset. Technical support issues arising from supporting information (other than missing files) should be addressed to the authors.

Supplementary
