# Supplementary material for: Electro‐Mechanochemical Atom Transfer Radical Cyclizations using Piezoelectric BaTiO3
Source: Angew Chem Int Ed Engl. 2020 Jul 9;59(38):16357–60. doi: 10.1002/anie.202003565 (PMC7540587; doi:10.1002/anie.202003565)

Angewandte Chemie
Eine Zeitschrift der Gesellschaft Deutscher Chemiker

Supporting Information

# Electro-Mechanochemical Atom Transfer Radical Cyclizations using Piezoelectric $BaTiO_3$

*Christian Schumacher, José G. Hernández,* and Carsten Bolm**

anie_202003565_sm_miscellaneous_information.pdf

**Supporting Information**

**Table of Contents**

-General information S2
-General procedure for the synthesis of starting materials; synthesis of secondary amines (procedure GP1A) S3
-General procedure for the synthesis of starting materials; synthesis of secondary amines (procedure GP1B) S3
-General procedure for the synthesis of starting materials; synthesis of tertiary amides (procedure GP2) S3
-Optimization of the ATRC of **1a** for the use of the piezoelectric effect S4
-ATRC of **1a** in the presence of piezoelectric and non-piezoelectric additives S5
-Influence of the number and size of milling balls on the ATRC of **1a** in the presence of $BaTiO_3$ S5
-Optimization of the milling time S6
-Control experiments S7
-Recyclability of $BaTiO_3$ S8
-ATRC of **1a** in the presence of $BaTiO_3$ in a planetary ball mill S8
-Effect of milling on *tet*- and *cub*-$BaTiO_3$ nanoparticle samples S9
-UV-Vis analysis S10
-General procedure for electro-mechanochemical ATRC of **1** using $BaTiO_3$ (procedure GP3) S10
-Analytical data of synthesized precursors and synthesized starting materials **1** S11
-Analytical data of ATRC products **2** S14
-References S15
-$^{1}H$ and $^{13}C\{^{1}H\}$ NMR spectra of new starting materials and ATRC products S16

## General Information

Mechanochemical reactions were performed either with a RETSCH Mixer Mill MM400 or a PULVERISETTE 7 premium line planetary mill. The milling containers and the milling balls used were always of the same material. To perform mechanochemical reactions under argon the milling containers were closed inside a glove box of the type MBraun Labmaster 130. Unless otherwise stated, all commercially available chemicals were used as received without further purification. The piezomaterials (*cub*-$BaTiO_3$, *tet*-$BaTiO_3$ and ZnO) that were used for this research project were purchased from US Research Nanomaterials, Inc. $Cu(OTf)_2$ (J&K Scientific 208060), TPMA (J&K Scientific 934562), $SrTiO_3$ (Aldrich 396141), $TiO_2$ (Aldrich 232033) and $Al_2O_3$ for chromatography, neutral, Brockmann I, 40-300 $\mu$m, 60A, mainly gamma-type aluminum oxide crystalline form according to the supplier (Acros Organics 366680025) were used as received. Solvents for flash column chromatography (FCC) purifications were of technical grade and were distilled before use. Flash column chromatography was conducted with Silica 60 M (0.04–0.063 mm) as stationary phase, which was purchased from MACHERY-NAGEL. Thin layer chromatography (TLC) was performed with silica coated alumina plates TLC Silica gel 60 $F_{254}$ from Merck and the products were visualized using UV-light ($\lambda$ = 254 nm) and/or by dipping the TLC plate in an aqueous solution of potassium permanganate ($KMnO_4$) and heating of the stained TLC plate with a heat gun until dryness. Alternatively, the products could be visualized by putting the TLC plates in an iodine chamber. Nuclear magnetic resonance (NMR) spectra were recorded either on a Varian Mercury 300, Varian VNMRS 400, Varian VNMRS 600, Bruker Avance Neo 400 or Bruker Avance Neo 600 at 25 °C, if not otherwise stated, and were processed and analyzed with the program MestReNova.[1] Chemical shifts ($\delta$) are given in parts per million (ppm). Proton and carbon NMR spectra were referenced to the non-deuterated residual solvent signal ($CHCl_3$: $^1$H NMR: $\delta$ = 7.26 ppm, $CDCl_3$: $^{13}C\{^1H\}$ NMR: $\delta$ = 77.16 ppm; toluene-$d_8$: $^1$H NMR: $\delta$ = 2.08, 6.97, 7.01, 7.09 ppm, toluene-$d_8$: $^{13}C\{^1H\}$ NMR: $\delta$ = 20.43, 125.13, 127.96, 128.87, 137.48 ppm).[2] Carbon spectra were measured proton broad band decoupled. The multiplicity of the peaks is reported as s (singlet), d (doublet), t (triplet), q (quartet), m (multiplet) and combinations thereof. The spin-spin coupling constants ($J$) are reported in Hertz (Hz). Infrared (IR) spectra were recorded neat on a PerkinElmer Spectrum 100 FT-IR spectrometer with an attached UATR device with a KRS-5 crystal for single reflection. IR bands are given with their corresponding wavenumber $\nu$ in $cm^{-1}$ and relative intensity of transmission (strong (s), medium (m), weak (w), broad (br)). Mass spectra were recorded on a Finnigan SSQ 7000 mass spectrometer [electron ionization (EI), 70 eV; chemical ionization (CI), methane, 100 eV]. The signals are given according to their *m/z* values and their relative intensity is reported in parenthesis. High resolution mass (HRMS) spectra were recorded as ESI (electrospray ionization, positive mode) on a ThermoFisher Scientific LTQ Orbitrap XL mass spectrometer. Powder X-ray diffraction (PXRD) pattern were recorded on a Bruker D2 PHASER X-ray diffractometer. The X-ray source was Copper-$K_\alpha$ radiation with a wavelength of $\lambda$ = 1.54056 Å. The spectra were recorded in a $2\Theta$ range of 15 to 70° and with a step size of $\Delta 2\Theta$ = 0.02029528°. UV–Vis spectra were measured on a Shimadzu UV-2600 spectrophotometer.

**General procedure for the synthesis of starting materials; synthesis of secondary amines (procedure GP1A)**

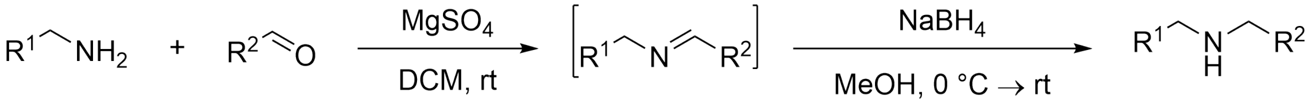


A round bottom flask of appropriate size equipped with a magnetic stirring bar was charged with 1.00 equiv. of the corresponding aldehyde, which was dissolved in DCM (0.5 M). Then, 1.00 equiv. of the corresponding amine and 1.00 equiv. of $MgSO_4$ were added and the reaction mixture was stirred at room temperature until monitoring the reaction by TLC indicated full conversion. The reaction mixture was filtered over a short plug of Celite and the solvent was evaporated under reduced pressure. The residue was dissolved in MeOH (0.25 M) and cooled to 0 °C in an ice bath. 1.50 equiv. of $NaBH_4$ were added in small portions. After the addition of reductant, the reaction mixture was allowed to warm up to room temperature overnight. The following day, the solvent was evaporated under reduced pressure, the residue was dissolved and partitioned between $H_2O$ and EtOAc. After phase separation, the aqueous phase was extracted three times with EtOAc. The organic phases were combined and dried over $Na_2SO_4$. If needed, the secondary amines were purified by flash column chromatography.

**General procedure for the synthesis of starting materials; synthesis of secondary amines (procedure GP1B)**

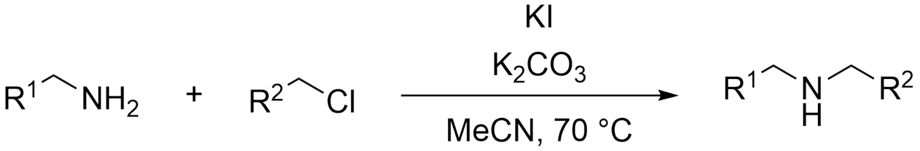


A round-bottom flask of appropriate size equipped with a magnetic stirring bar was charged with 1.00 equiv. of the corresponding alkenyl chloride, which was dissolved in MeCN (0.2 M). Then, 0.10 equiv. of potassium iodide were added, and the reaction mixture was stirred at room temperature for 1 h. Next, 2.00 equiv. of $K_2CO_3$ and 1.00 equiv. of the corresponding primary amine were added, and the reaction mixture was stirred at 70 °C in an oil bath until monitoring of the reaction by TLC indicated full conversion. The solvent was evaporated, and the secondary amines were purified by flash column chromatography.

**General procedure for the synthesis of starting materials; synthesis of tertiary amides (procedure GP2)**

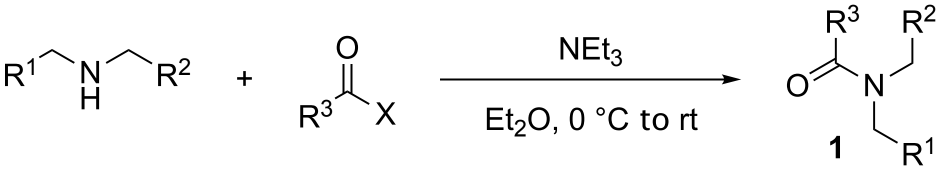


A round-bottom flask of appropriate size was equipped with a magnetic stirring bar and charged with the corresponding secondary amine (1.00 equiv.). The amine was dissolved in distilled $Et_2O$ (0.1-0.5 M) and cooled to 0 °C in an ice bath. Then, 2.00 equiv. of $NEt_3$ were added followed by slow addition of the corresponding acyl halide (1.00 equiv.). After the addition, the reaction mixture was allowed to warm up to room temperature overnight. The following day, the reaction was stopped by adding an aqueous saturated $NH_4Cl$ solution. The biphasic reaction mixture was stirred vigorously for 15 minutes. Then, it was partitioned between an aqueous saturated $NaHCO_3$ solution and distilled $Et_2O$. The aqueous phase was extracted three times with distilled $Et_2O$, the organic phases were combined and

dried over $Na_2SO_4$. The solvent was evaporated, and the amides **1** were purified by flash column chromatography.

**Optimization of the ATRC of 1a for the use of the piezoelectric effect**

A 10 mL $ZrO_2$ milling vessel equipped with one $ZrO_2$ milling ball (10 mm in diameter) was charged with *N*-allyl-*N*-benzyl-2-bromo-2-methylpropanamide (**1a**, 100 mg, 0.34 mmol, 1.00 equiv.). Then, the jar was transferred to the glove box and the corresponding Cu-salt and/or ligand were added in the given order. The jar was closed and sealed with parafilm inside the glove box. After transferring the milling vessel out of the glove box the mechanochemical reaction was carried out for 90 min at 25 Hz. After milling, $CDCl_3$ (1 mL) was added to the jar, which was closed and shaken. The resulting reaction mixture was filtered over a short plug of silica using a pipette filter. The filtrate was analyzed by $^1H$ NMR spectroscopy. The corresponding results are listed in Table S1.

**Table S1.** ATRC reaction of **1a** without the use of piezoelectric materials.

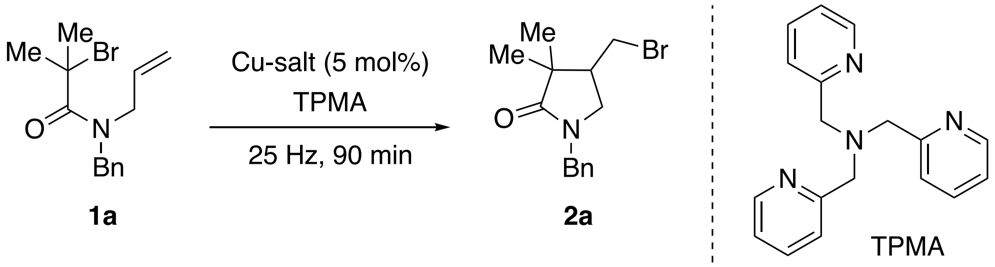


**1a** **2a**

| Cu-salt | TPMA [mol%] | **1a**:**2a** [%][a] |
|---|---|---|
| $Cu(OTf)_2$ | 15 | 0:100 |
| $Cu(OTf)_2$ | 4.5 | 97:3 |
| $Cu(OTf)_2$ | 2.5 | 100:0 |
| – | 30 | 100:0 |
| $Cu(OTf)_2$ | – | 100:0 |
| CuBr | – | 100:0 |
| CuBr | 5 | 10:90 |
| $CuBr_2$ | 5 | 100:0 |
| $CuBr_2$ | 15 | 84:16 |

[a] Determined by $^1H$ NMR spectroscopy.

**ATRC of 1a in the presence of piezoelectric and non-piezoelectric additives**

A 10 mL $ZrO_2$ milling vessel equipped with one $ZrO_2$ milling ball (10 mm in diameter) was charged with *N*-allyl-*N*-benzyl-2-bromo-2-methylpropanamide (**1a**, 100 mg, 0.34 mmol, 1.00 equiv.). Then, the jar was transferred to the glove box and $Cu(OTf)_2$ (6.4 mg, 0.017 mmol, 5 mol%), TPMA (4.4 mg, 0.015 mmol, 4.5 mol%) and the corresponding additive (see Table 2 in the paper) were added in the given order. The jar was closed and sealed with parafilm inside the glove box. After transferring the milling vessel out of the glove box, the mechanochemical reaction was carried out for 90 minutes at 25 Hz. After milling, $CDCl_3$ (1 mL) was added to the jar, which was closed and shaken. The resulting reaction mixture was filtered over a short plug of silica using a pipette filter. The filtrate was analyzed by $^1H$ NMR spectroscopy.

**Influence of the number and size of milling balls on the ATRC of 1a in the presence of $BaTiO_3$**

A 10 mL $ZrO_2$ milling vessel equipped with the chosen amount of $ZrO_2$ milling balls (3-10 mm in diameter) was charged with *N*-allyl-*N*-benzyl-2-bromo-2-methylpropanamide (**1a**, 100 mg, 0.34 mmol, 1.00 equiv.). Then, the jar was transferred to the glove box and $Cu(OTf)_2$ (6.1 mg, 0.017 mmol, 5 mol%), TPMA (4.4 mg, 0.015 mmol, 4.5 mol%) and $BaTiO_3$ (tetragonal, 500 nm, 40 wt%) were added in the given order. The jar was closed and sealed with parafilm inside the glove box. After transferring the milling vessel out of the glove box, the mechanochemical reaction was carried out for 90 minutes at 25 Hz. After milling, $CDCl_3$ (1 mL) was added to the jar, which was closed and shaken. The resulting reaction mixture was filtered over a short plug of silica using a pipette filter. The filtrate was analyzed by $^1H$ NMR spectroscopy. The corresponding results are listed in Table S2. All the experiments were done in quadruplicate.

**Table S2.** Results of the influence of ball amounts and ball size.

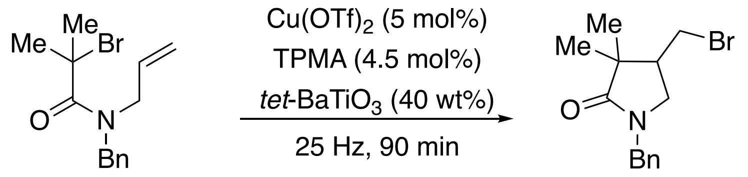


**1a** **2a**

| Diameter of ball [mm] | Number of balls | Total mass of the balls [g] | **1a**:**2a** [%][a] |
|---|---|---|---|
| 10 | 1 | 3.4509 | 68:32 |
| 5 | 8 | 3.3300 | 3:97 |
| 3 | 39 | 3.4075 | 60:40 |

[a] Determined by $^1H$ NMR spectroscopy.

**Optimization of the milling time**

A 10 mL $ZrO_2$ milling vessel equipped with eight $ZrO_2$ milling balls (5 mm in diameter) was charged with *N*-allyl-*N*-benzyl-2-bromo-2-methylpropanamide (**1a**, 100 mg, 0.34 mmol, 1.00 equiv.). Then, the jar was transferred to the glove box and $Cu(OTf)_2$ (6.1 mg, 0.017 mmol, 5 mol%), TPMA (4.4 mg, 0.015 mmol, 4.5 mol%) and $BaTiO_3$ (tetragonal, 500 nm, 20 wt%) were added in the given order. The jar was closed and sealed with parafilm inside the glove box. After transferring the milling vessel out of the glove box, the mechanochemical reaction was carried out for the chosen amount of time at 25 Hz. After milling, $CDCl_3$ (1 mL) was added to the jar, which was closed and shaken. The resulting reaction mixture was filtered over a short plug of silica using a pipette filter. The filtrate was analyzed by $^1H$ NMR spectroscopy. The corresponding results are listed in Table S3.

**Table S3.** Results of the milling time optimization.

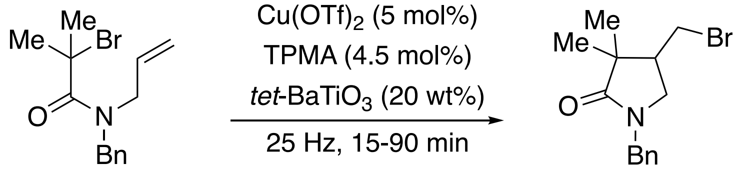


**1a** **2a**

| Milling Time [min] | **1a**:**2a** [%][a] | **1a**:**2a** [%][a] Average |
|---|---|---|
| 15 | Repetition (1) 89:11 | 95:5 |
| | Repetition (2) 97:3 | |
| | Repetition (3) 93:7 | |
| | Repetition (4) 100:0 | |
| 30 | Repetition (1) 100:0 | 98:2 |
| | Repetition (2) 100:0 | |
| | Repetition (3) 100:0 | |
| | Repetition (4) 92:8 | |
| 60 | Repetition (1) 46:54 | 66:34 |
| | Repetition (2) 81:19 | |
| | Repetition (3) 100:0 | |
| | Repetition (4) 38:62 | |
| 90 | Repetition (1) 2:98 | 1:99 |
| | Repetition (2) 0:100 | |
| | Repetition (3) 0:100 | |
| | Repetition (4) 0:100 | |

[a] Determined by $^1H$ NMR spectroscopy.

**Control experiments**

A 10 mL $ZrO_2$ milling vessel equipped with eight $ZrO_2$ milling balls (5 mm in diameter) was charged with *N*-allyl-*N*-benzyl-2-bromo-2-methylpropanamide (**1a**, 100 mg, 0.34 mmol, 1.00 equiv.). Then, the jar was transferred to the glove box and if used $Cu(OTf)_2$ (6.1 mg, 0.017 mmol, 5 mol%) and/or TPMA (4.4 mg, 0.015 mmol, 4.5 mol%) and/or $BaTiO_3$ (tetragonal, 500 nm) were added in the given order. The jar was closed and sealed with parafilm inside the glove box. After transferring the milling vessel out of the glove box, the mechanochemical reaction was carried out for 90 minutes at 25 Hz. After milling, $CDCl_3$ (1 mL) was added to the jar, which was closed and shaken. The resulting reaction mixture was filtered over a short plug of silica using a pipette filter. The filtrate was analyzed by $^1H$ NMR spectroscopy. The corresponding results are listed in Table S4.

**Table S4.** Results of control experiments.

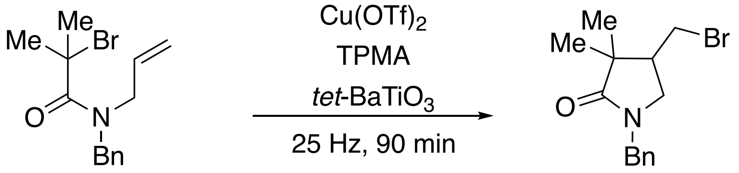


**1a** **2a**

<table>
<tr><th>Cu(OTf)<sub>2</sub> [mol%]</th><th>TPMA [mol%]</th><th>tet-BaTiO<sub>3</sub> [wt%]</th><th colspan="2">1a:2a [%][a]</th></tr>
<tr><td>5</td><td>4.5</td><td>20</td><td colspan="2">3:97</td></tr>
<tr><td>5</td><td>–</td><td>–</td><td colspan="2">100:0</td></tr>
<tr><td>–</td><td>4.5</td><td>–</td><td colspan="2">100:0</td></tr>
<tr><td rowspan="4">5</td><td rowspan="4">4.5</td><td rowspan="4">–</td><td>Repetition (1)<br>1a:2a 100:0</td><td rowspan="4">Average<br>1a:2a<br>79:21</td></tr>
<tr><td>Repetition (2)<br>1a:2a 100:0</td></tr>
<tr><td>Repetition (3)<br>1a:2a 100:0</td></tr>
<tr><td>Repetition (4)<br>1a:2a 16:84</td></tr>
</table>

[a] Determined by $^1H$ NMR spectroscopy.

**Recyclability of $BaTiO_3$**

A 10 mL $ZrO_2$ milling vessel equipped with eight $ZrO_2$ milling balls (5 mm in diameter) was charged with *N*-allyl-*N*-benzyl-2-bromo-2-methylpropanamide (**1a**, 100 mg, 0.34 mmol, 1.00 equiv.). Then, the jar was transferred to the glove box and $Cu(OTf)_2$ (6.1 mg, 0.017 mmol, 5 mol%), TPMA (4.4 mg, 0.015 mmol, 4.5 mol%) and $BaTiO_3$ (tetragonal, 500 nm, 20 wt%) were added in the given order. The jar was closed and sealed with parafilm inside the glove box. After transferring the milling vessel out of the glove box, the mechanochemical reaction was carried out for 90 minutes at 25 Hz. After milling, $CDCl_3$ (1.2 mL) was added to the jar, which was closed and shaken. The resulting reaction mixture was centrifuged (3 min, 5000 rpm). The supernatant solution was analyzed by $^1H$ NMR spectroscopy. The solid residue was transferred in a round-bottom flask using DCM. The solvent was evaporated, and the residue suspended in MeOH. The suspension was filtered through a fritted funnel and the solid was washed with MeOH. The resulting white powder was dried in a stream of air and tested in the ATRC of **1a**. The corresponding results are listed in Table S5.

**Table S5.** Recyclability of $BaTiO_3$ in the ATRC of **1a**.

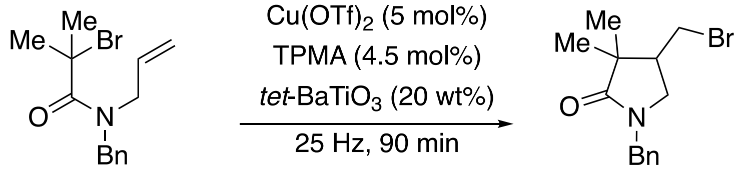


**1a** **2a**

| $BaTiO_3$ | **1a**:**2a** [%][a] |
|---|---|
| New (tetragonal, 500 nm) | 17:83 |
| Recycled (tetragonal, 500 nm) | 18:82 |

[a] Determined by $^1H$ NMR spectroscopy.

**ATRC of 1a in the presence of $BaTiO_3$ in a planetary ball mill**

A 20 mL $ZrO_2$ milling vessel equipped with eight $ZrO_2$ milling balls (5 mm in diameter) was charged with *N*-allyl-*N*-benzyl-2-bromo-2-methylpropanamide (**1a**, 100 mg, 0.34 mmol, 1.00 equiv.). Then, the jar was transferred to the glove box and $Cu(OTf)_2$ (6.1 mg, 0.017 mmol, 5 mol%), TPMA (4.4 mg, 0.015 mmol, 4.5 mol%) and $BaTiO_3$ (tetragonal, 500 nm, 20 wt%) were added in the given order. The jar was closed in the glove box. After transferring the milling vessel out of the glove box, the mechanochemical reaction was carried out for 90 minutes at the chosen frequency. After milling, $CDCl_3$ (1 mL) was added to the jar, which was closed and shaken. The resulting reaction mixture was filtered over a short plug of silica using a pipette filter. The filtrate was analyzed by $^1H$ NMR spectroscopy revealing only the presence of **1a**.

**Table S6.** ATRC of **1a** in the presence of $BaTiO_3$ in a planetary ball mill.

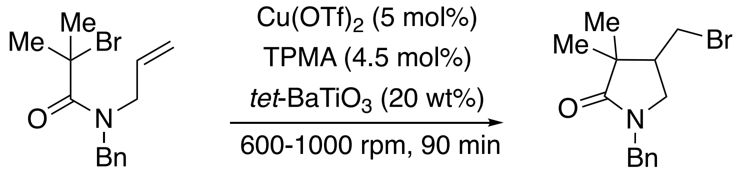


**1a** **2a**

| Milling Frequency [rpm] | **1a**:**2a** [%][a] | **1a**:**2a** [%][a] Average |
|---|---|---|
| 600 | Repetition (1) 99:1 | 99:1 |
| | Repetition (2) 99:1 | |
| 800 | Repetition (1) 89:1 | 89:11 |
| | Repetition (2) 89:11 | |
| 1000 | Repetition (1) 100:0 | 82:18 |
| | Repetition (2) 64:36 | |

[a] Determined by $^{1}H$ NMR spectroscopy.

**Effect of milling on *tet*- and *cub*-$BaTiO_3$ nanoparticles**

A 10 mL $ZrO_2$ milling vessel equipped with eight $ZrO_2$ milling balls (5 mm in diameter) was charged approximately with 200 mg of $BaTiO_3$ of the chosen phase inside a glove box and was sealed with parafilm. Then, the milling vessel was transferred outside the glove box and the sample was milled for 90 minutes at 25 Hz. After milling, the sample was scratched out of the jar using a single use spatula and was analyzed by PXRD (Figure S1).

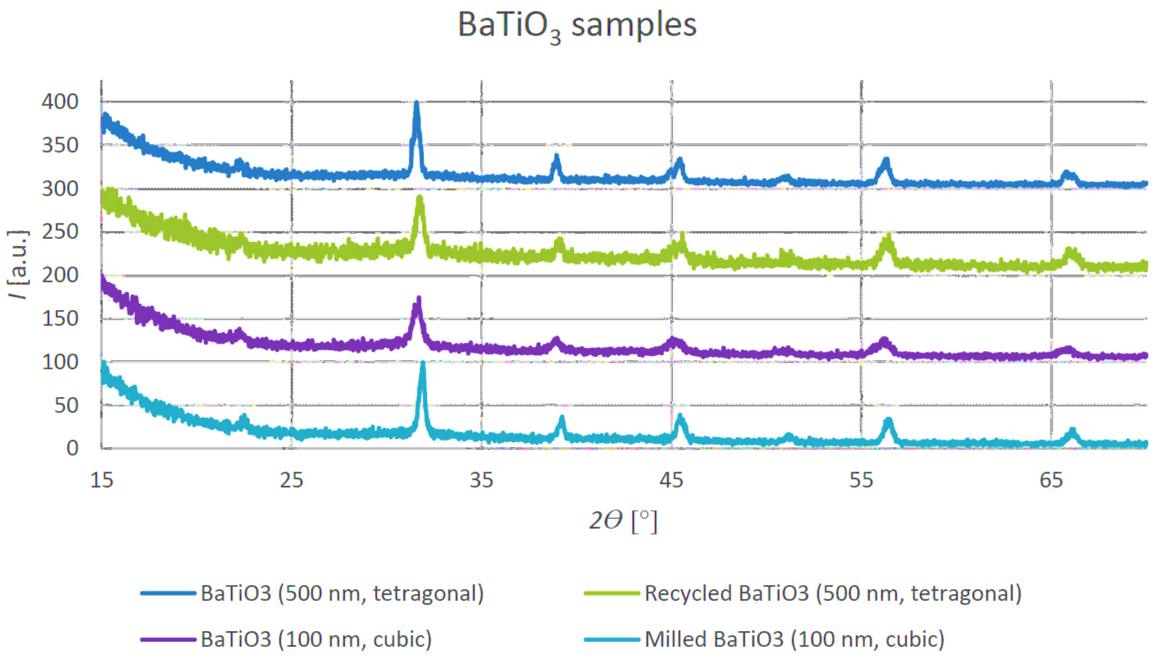


**Figure S1**. PXRD patterns of $BaTiO_3$ samples.

## UV-Vis analysis

A 10 mL $ZrO_2$ milling vessel equipped with eight $ZrO_2$ milling balls (5 mm in diameter) was charged with $Cu(OTf)_2$ (100 mg, 0.276 mmol), TPMA (72.3 mg, 0.249 mmol) and *tet*-$BaTiO_3$ (34.5 mg) or $Al_2O_3$ (34.5 mg) inside a glove box. Then, the milling jars were taken out of the glove box and their content were milled for 90 minutes at 25 Hz. After this time, the milling jars were opened inside the glove box. A determined amount of the samples was transferred to Schlenk flasks and DMSO (3.0 mL) was added. The obtained reaction mixture was filtered under inert atmosphere over a short plug of Celite using a pipette filter. The resulting solution was transferred to a UV-Vis cuvette which was stored in a Schlenk tube and was evacuated and flushed with argon (3 cycles). Next, the UV-Vis spectra of the DMSO solutions under anaerobic conditions were measured (Figure S2).

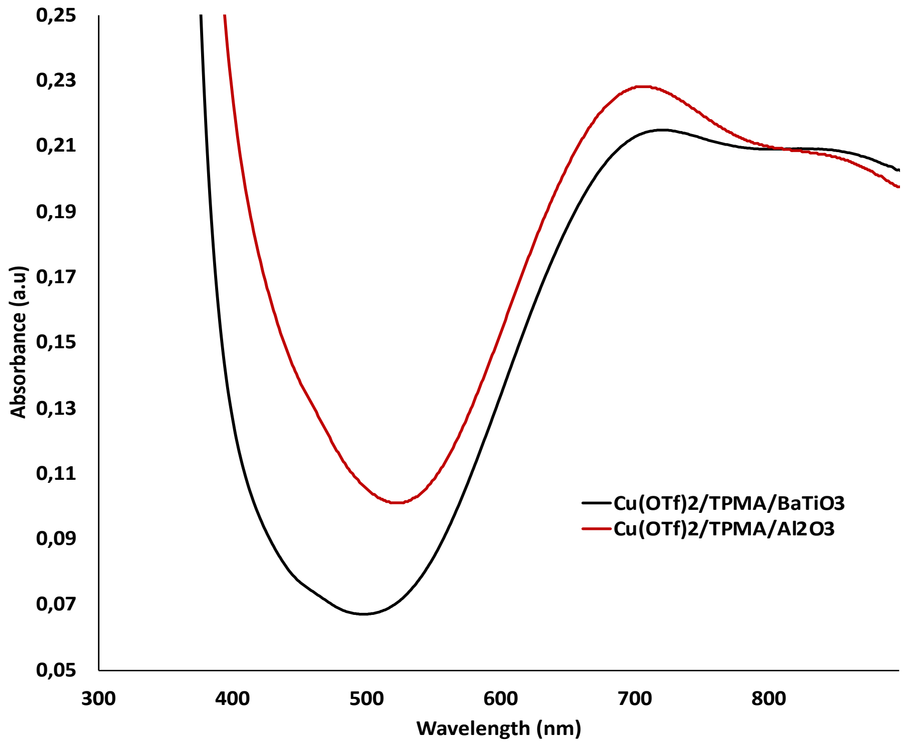


**Figure S2**. UV-Vis spectra of solutions in DMSO from milling experiments of $Cu(OTf)_2$, TPMA and *tet*-$BaTiO_3$ or $Al_2O_3$.

## General procedure for electro-mechanochemical ATRC of 1 using $BaTiO_3$ (procedure GP3)

A 10 mL $ZrO_2$ milling vessel equipped with eight $ZrO_2$ milling balls (5 mm in diameter) was charged with 0.34 mmol of the corresponding amide **1**. Then, the jar was transferred to the glove box and $Cu(OTf)_2$ (6.1 mg, 0.017 mmol, 5 mol%), TPMA (4.4 mg, 0.015 mmol, 4.5 mol%) and $BaTiO_3$ (tetragonal, 500 nm, 20 wt%) were added in the given order. The jar was closed and sealed with parafilm inside the glove box. After transferring the milling vessel out of the glove box, the mechanochemical reaction was carried out for 90 minutes (or 180 minutes) at 25 Hz. After milling, the reaction mixture was transferred in a flask by conducting five repetitions of adding $CHCl_3$ (5 mL) to the jar, closing and shaking it and pouring the reaction mixture into the flask. A small amount of silica was added, and the solvent was evaporated. Products **2** were purified by column chromatography.

**Analytical data of synthesized precursors and synthesized starting materials 1**

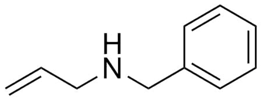

*N*-Allyl-*N*-benzylamine: The title compound was synthesized using general procedure GP1A and was obtained as a slightly yellow oil (6.2966 g, 42.77 mmol, 86%) after column chromatography ($SiO_2$/acetone). ***R*f** = 0.63 (acetone), UV-active, stains with $I_2$; **$^1$H NMR (400 MHz, $CDCl_3$)**: $\delta$ = 7.37–7.21 (m, 5H, Ar–*H*), 5.94 (ddt, $^3J_{H,H}$ = 17.3, 10.3, 6.0 Hz, 1H, RC*H*=$CH_2$), 5.20 (dq, $^3J_{H,H}$ = 17.1, 1.6 Hz, 1H, RCH=C$H_AH_B$), 5.12 (dq, $^3J_{H,H}$ = 10.3, 1.4 Hz, 1H, RCH=C$H_AH_B$), 3.80 (s, 2H, PhC$H_2$NHR), 3.28 (dt, $^3J_{H,H}$ = 6.0, 1.5 Hz, 2H, RNHC$H_2$CH=$CH_2$), 1.45 (br s, 1H, N*H*) ppm; **$^{13}$C{$^1$H} NMR (101 MHz, $CDCl_3$)**: $\delta$ = 140.4, 136.9, 128.5, 128.3, 127.1, 116.1, 53.4, 51.9 ppm; **IR (ATR)**: $\nu$ = 3855 (w), 3653 (w), 3315 (br w), 3071 (m), 3027 (m), 2913 (w), 2811 (m), 2331 (br w), 2088 (w), 1993 (w), 1870 (w), 1738 (w), 1641 (m), 1601 (w), 1492 (m), 1451 (s), 1357 (w), 1243 (w), 1201 (w), 1106 (s), 1028 (w), 992 (m), 916 (s), 820 (w), 734 (s), 698 (s) $cm^{-1}$; **MS (100 eV, CI)**: *m*/*z* (%): 148 (28) $[M+H]^+$; **MS (70 eV, EI)**: *m*/*z* (%): 147 (25) $[M]^+$, 146 (72) $[M-H]^+$, 120 (11), 91 (100) $[C_7H_7]^+$. The NMR data closely match the ones previously reported in the literature.[3]

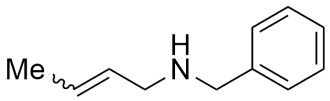

*N*-Crotylbenzylamine: The title compound was synthesized using general procedure GP1B and was obtained as a mixture of *cis* and *trans* isomer as a yellow oil (0.3870 g, 2.40 mmol, 24%) after column chromatography ($SiO_2$/*n*-Pentane:EtOAc 95:5 *v*/*v* → EtOAc). ***R*f** = 0.13 (EtOAc), smears, UV-active, stains with $KMnO_4$; **$^1$H NMR (400 MHz, $CDCl_3$)**: $\delta$ = 7.35–7.21 (m, 5H, Ar–*H*), 5.59 (m, 2H, RC*H*=C*H*R), 3.80 (s, 0.38H, PhC$H_2$ (*cis*)), 3.78 (s, 1.82H, PhC$H_2$ (*trans*)), 3.31 (m, 0.38H, RNHC$H_2$R (*cis*)), 3.21 (m, 1.82H, RNHC$H_2$R (*trans*)), 1.70 (m, 2.73H, RC*H*=CHC$H_3$ (*trans*)), 1.63 (m, 0.27H, RC*H*=CHC$H_3$ (*cis*)), 1.47 (br s, 1H, N*H*) ppm; **$^{13}$C{$^1$H} NMR (101 MHz, $CDCl_3$ (*trans* only))**: $\delta$ = 140.5, 129.6, 128.5, 128.3, 127.6, 127.0, 53.4, 51.3, 17.9 ppm; **IR (ATR)**: $\nu$ = 3313 (br w), 3063 (w), 3025 (m), 2918 (m), 2812 (m), 2333 (br w), 2116 (w), 2044 (w), 1947 (w), 1873 (w), 1808 (w), 1670 (w), 1601 (w), 1541 (w), 1493 (m), 1449 (s), 1361 (m), 1296 (w), 1200 (w), 1112 (m), 1064 (w), 1027 (w), 966 (s), 907 (m), 822 (w), 732 (s), 697 (s) $cm^{-1}$; **MS (100 eV, CI)**: *m*/*z* (%): 162 (76) $[M+H]^+$; **MS (70 eV, EI)**: *m*/*z* (%): 161 (40) $[M]^+$, 160 (17) $[M-H]^+$, 146 (19), 106 (30), 91 (100) $[C_7H_7]^+$, 70 (16), 65 (14), 55 (13). The NMR data closely match the ones previously reported in the literature.[3]

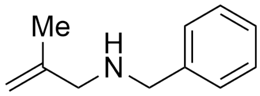

*N*-(2-Methallyl)benzylamine: The title compound was synthesized using general procedure GP1B and was obtained as a slightly yellow oil (0.8362 g, 5.19 mmol, 52%) after column chromatography ($SiO_2$/*n*-Pentane:EtOAc 95:5 *v*/*v* → EtOAc). ***R*f** = 0.29 (EtOAc), smears, UV-active, stains with $KMnO_4$; **$^1$H NMR (600 MHz, $CDCl_3$)**: $\delta$ = 7.39–7.29 (m, 4H, Ar–*H*), 7.28-7.23 (m, 1H, Ar–*H*), 4.91 (s, 1H, RC($CH_3$)=C$H_AH_B$), 4.86 (s, 1H, RC($CH_3$)=C$H_AH_B$), 3.77 (s, 2H, NC$H_2$C=$CH_2$), 3.20 (s, 2H, PhC$H_2$NH), 1.77 (s, 3H, C$H_3$), 1.52 (br s, 1H, N*H*) ppm; **$^{13}$C{$^1$H} NMR (151 MHz, $CDCl_3$)**: $\delta$ = 144.0, 140.6, 128.5, 128.3, 127.0, 110.9, 55.2, 53.2, 20.9 ppm; **IR (ATR)**: $\nu$ = 3868 (br w), 3651 (w), 3329 (w), 3071 (m), 3029 (w), 2971 (w), 2910 (m), 2817 (m), 2332 (br w), 2097 (w), 1944 (w), 1873 (w), 1801 (w), 1651 (m), 1598 (w), 1449 (s), 1364 (m), 1235 (w), 1200 (w), 1114 (s), 1027 (w), 971 (w), 893 (s), 822 (w), 734 (s), 699 (s) $cm^{-1}$; **MS (100 eV, CI)**: *m*/*z* (%): 162 (100) $[M+H]^+$; **MS (70 eV, EI)**: *m*/*z* (%): 161 (21) $[M]^+$, 160 (43) $[M-H]^+$, 146 (11), 120 (17), 106 (9), 91 (100) $[C_7H_7]^+$. The NMR data closely match the ones previously reported in the literature.[3]

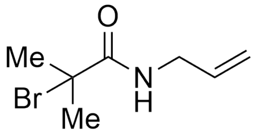

*N*-Allyl-2-bromo-2-methylpropanamide (**1a-NH**): The title compound was synthesized using general procedure GP2 and was obtained as a colorless oil (3.1250 g, 15.16 mmol, 91%) after column chromatography ($SiO_2$/*n*-Pentane:EtOAc 4:1 *v/v*). ***R*<sub></sub>** $R_f$ = 0.49 (*n*-Pentane:EtOAc 4:1 *v/v*), UV-active, stains with $KMnO_4$; **$^1$H NMR (600 MHz, $CDCl_3$)**: $\delta$ = 6.80 (br s, 1H, N*H*), 5.86 (m, 1H, RC*H*=$CH_2$), 5.23 (m, 1H; RCH=C$H_A$H$_B$), 5.17 (m, 1H, RCH=CH$_A$$H_B$), 3.89 (tt, $^3J_{H,H}$ = 5.7, 1.6 Hz, 2H, RC$H_2$CH), 1.97 (s, 6H, 2×C$H_3$) ppm; **$^{13}$C{$^1$H} NMR (151 MHz, $CDCl_3$)**: $\delta$ = 172.0, 133.7, 116.7, 63.3, 42.8, 32.8 ppm; **IR (ATR)**: $\nu$ = 3880 (br w), 3342 (s), 3079 (w), 2982 (w), 2923 (w), 2664 (w), 2324 (br w), 2105 (w), 1994 (w), 1862 (br w), 1654 (s), 1524 (s), 1423 (w), 1368 (w), 1277 (m), 1190 (m), 1111 (s), 991 (m), 918 (s), 828 (w), 767 (w), 666 (w) cm$^{-1}$; **MS (100 eV, CI)**: *m*/*z* (%): 206 (100) [*M*+H]$^+$; **MS (70 eV, EI)**: *m*/*z* (%): 206 (43) [*M*+H]$^+$, 126 (100), 84 (20). The NMR data closely match the ones previously reported in the literature.[4]

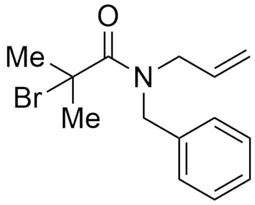

*N*-Allyl-*N*-benzyl-2-bromo-2-methylpropanamide (**1a**): The title compound was synthesized using general procedure GP2 and was obtained as a slightly yellow oil (11.2942 g, 38.13 mmol, 94%) after column chromatography ($SiO_2$/*n*-Pentane:EtOAc 4:1 *v/v*). $R_f$ = 0.84 (*n*-Pentane:EtOAc 1:1 *v/v*), UV-active, stains with $KMnO_4$; **$^1$H NMR (400 MHz, toluene-*d*$_8$, 95 °C)**: $\delta$ = 7.19–6.93 (m, 5H, Ar–*H*), 5.60 (m, 1H, RC*H*=$CH_2$), 4.94 (m, 2H, RCH=C$H_2$), 4.65 (s, 2H, NC$H_2$Ph), 4.00 (s, 2H, NC$H_2$), 1.82 (s, 6H, 2×C$H_3$) ppm; **$^{13}$C{$^1$H} NMR (101 MHz, toluene-*d*$_8$, 95 °C)**: $\delta$= 170.3, 137.6, 133.8, 128.9, 128.0, 127.5, 117.4, 57.9, 50.7, 50.4, 33.2 ppm; **IR (ATR)**: $\nu$ = 3261 (w), 3066 (w), 3012 (w), 2980 (w), 2930 (w), 2662 (w), 2334 (br w), 2195 (w), 2098 (br w), 1950 (w), 1635 (s), 1494 (w), 1460 (m), 1411 (s), 1364 (m), 1285 (w), 1247 (m), 1169 (s), 1108 (s), 1026 (w), 996 (m), 927 (m), 808 (w), 730 (m), 698 (m) cm$^{-1}$; **MS (100 eV, CI)**: *m*/*z* (%): 296 (91) [*M*+H]$^+$; **MS (70 eV, EI): *m*/*z* (%)**: 296 (12) [*M*+H]$^+$, 216 (100), 91 (43) [$C_7H_7$]$^+$. The NMR data closely match the ones previously reported in the literature.[5]

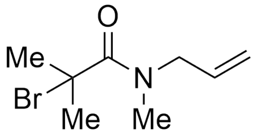

*N*-Allyl-2-bromo-*N*-2-dimethylpropanamide (**1b**): The title compound was synthesized using general procedure GP2 and was obtained as a colorless oil (2.5796 g, 11.72 mmol, 84%) after column chromatography ($SiO_2$/*n*-Pentane:EtOAc 4:1 *v/v*). $R_f$ = 0.37 (*n*-Pentane:EtOAc 4:1 *v/v*), UV-active, stains with $KMnO_4$; **$^1$H NMR (600 MHz, toluene-*d*$_8$, 95 °C)**: $\delta$= 5.58 (m, 1H, RC*H*=$CH_2$), 4.96 (m, 1H, RCH=C$H_A$H$_B$), 4.93 (m, 1H, RCH=CH$_A$$H_B$), 3.88 (d, $^3J_{H,H}$ = 5.7 Hz, 2H, NC$H_2$), 2.82 (s, 3H, NC$H_3$), 1.78 (s, 6H, 2×C$H_3$) ppm; **$^{13}$C{$^1$H} NMR (151 MHz, toluene-*d*$_8$. 95 °C)**: $\delta$= 169.9, 133.9, 116.9, 57.7, 53.4, 36.4, 33.0, 20.4 ppm; **IR (ATR)**: $\nu$= 3260 (w), 3082 (w), 2978 (w), 2929 (w), 2161 (w), 2017 (w), 1632 (s), 1467 (m), 1426 (m), 1395 (s), 1371 (m), 1280 (m), 1178 (m), 1087 (s), 988 (m), 923 (m), 756 (w) cm$^{-1}$; **MS (100 eV, CI)**: *m*/*z* (%): 220 (59) [*M*+H]$^+$; **MS (70 eV, EI)**: *m*/*z* (%): 220 (30) [*M*+H]$^+$, 219 (14) [*M*]$^+$, 140 (100), 98 (58), 70 (21); **HRMS (ESI)**: *m*/*z* calculated for $C_8H_{14}BrNO+Na^+$: 242.0151 [*M*+Na]$^+$; found: 242.0152.

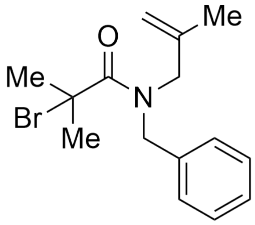

*N*-Benzyl-2-bromo-2-methyl-*N*-(2-methylallyl)propanamide (**1c**): The title compound was synthesized using general procedure GP2 and was obtained as a slightly yellow oil (1.4818 g, 4.78 mmol, 93%) after column chromatography ($SiO_2$/*n*-Pentane:EtOAc 19:1 *v/v*). $R_f$ = 0.35 (*n*-Pentane:EtOAc 19:1 *v/v*), UV-active, stains with $KMnO_4$; **$^1$H NMR (600 MHz, toluene-*d*$_8$, 95 °C)**: $\delta$= 7.14–6.93 (m, 5H, Ar–*H*), 4.78 (s, 1H, R=C$H_A$H$_B$), 4.70 (s, 2H, PhC$H_2$N), 4.68 (s, 1H, R=CH$_A$$H_B$), 4.01 (s, 2H, NCH$_2$), 1.83 (s, 6H, 2×C$H_3$), 1.51 (s, 3H, CH$_2$C$H_3$=CH$_2$) ppm; **$^{13}$C{$^1$H} NMR (151 MHz, toluene-*d*$_8$, 95 °C)**: $\delta$= 170.6, 141.1, 138.1, 128.9, 128.0, 127.6, 112.4, 58.2, 53.0, 51.2, 33.4, 20.0 ppm; **IR (ATR)**: $\nu$ = 3264 (w), 3068 (w), 2976 (w), 2931 (w), 2327 (br w),

2094 (w), 1994 (w), 1882 (br w), 1637 (s), 1494 (w), 1457 (m), 1414 (s), 1369 (m), 1290 (w), 1247 (m), 1168 (s), 1108 (m), 1079 (w), 1053 (w), 1029 (w), 1001 (w), 939 (w), 896 (m), 819 (w), 731 (s), 698 (m) cm$^{-1}$; **MS (100 eV, CI)**: *m*/*z* (%): 310 (4) [*M*+H]$^+$; **MS (70 eV, EI)**: *m*/*z* (%): 310 (14) [*M*+H]$^+$, 309 (20) [*M*]$^+$, 230 (100), 160 (15), 91 (46) [$C_7H_7$]$^+$; **HRMS (ESI)**: *m*/*z* calculated for $C_{15}H_{20}BrNO+Na^+$: 332.0621 [*M*+Na]$^+$; found: 332.0621.

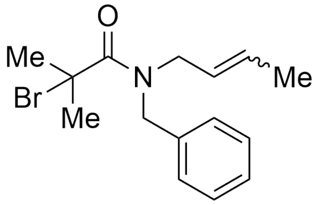

*N*-Benzyl-2-bromo-*N*-crotyl-2-methylpropanamide (**1d**): The title compound was synthesized using general procedure GP2 and was obtained as a yellow oil (0.6453 g, 2.08 mmol, 92%) after column chromatography ($SiO_2$/*n*-Pentane:EtOAc 19:1 *v/v*). ***R*f** = 0.32 (*n*-Pentane:EtOAc 19:1 *v/v*), UV-active, stains with $KMnO_4$; **$^1$H NMR (400 MHz, toluene-*d*8, 95 °C)**: $\delta$ = 7.16–6.90 (m, 5H, Ar–*H*), 5.33 (m, 2H, RC*H*=C*H*CH$_3$), 4.66 (m, 2H, NC*H*$_2$Ph), 3.99 (m, 2H, NC*H*$_2$), 1.84 (m, 6H, 2×C*H*$_3$), 1.48 (m, 3H, CHC*H*$_3$) ppm; **$^{13}$C{$^1$H} NMR (101 MHz, toluene-*d*8, 95 °C)**: $\delta$ = 170.3, 138.3, 128.9, 128.1, 127.5, 126.9, 58.0, 50.5, 50.0, 33.3, 17.5 ppm; **IR (ATR)**: $\nu$ = 3885 (br w), 3260 (w), 3008 (w), 2974 (w), 2930 (m), 2328 (br w), 2074 (w), 2011 (w), 1874 (br w), 1634 (s), 1494 (w), 1415 (s), 1363 (m), 1244 (m), 1167 (s), 1108 (s), 1026 (w), 1001 (m), 966 (m), 900 (w), 813 (w), 728 (m), 698 (m) cm$^{-1}$; **MS (100 eV, CI)**: *m*/*z* (%): 310 (92) [*M*+H]$^+$; **MS (70 eV, EI)**: *m*/*z* (%): 310 (22) [*M*+H]$^+$, 230 (100), 91 (73) [$C_7H_7$]$^+$, 55 (22); **HRMS (ESI)**: *m*/*z* calculated for $C_{15}H_{20}BrNO+Na^+$: 332.0621 [*M*+Na]$^+$; found: 332.0620.

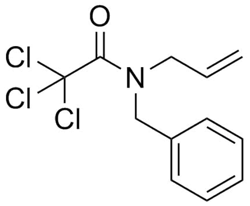

*N*-Allyl-*N*-benzyl-2,2,2-trichloroacetamide (**1e**): The title compound was synthesized using general procedure GP2 and was obtained as a colorless oil (2.7234 g, 9.31 mmol, 93%) after column chromatography ($SiO_2$/*n*-Pentane:EtOAc 4:1 *v/v*). ***R*f** = 0.76 (*n*-Pentane:EtOAc 4:1 *v/v*), UV-active, stains with $KMnO_4$; **$^1$H NMR (600 MHz, toluene-*d*8, 95 °C)**: $\delta$ = 7.12–6.93 (m, 5H, Ar–*H*), 5.53 (m, 1H, RC*H*=CH$_2$), 4.95 (d, $^3J_{H,H}$ = 10.3 Hz, 1H, RCH=C*H*$_A$CH$_B$), 4.88 (d, $^3J_{H,H}$ = 17.3 Hz, 1H, RCH=CH$_A$C*H*$_B$), 4.56 (s, 2H, PhC*H*$_2$N), 3.90 (s, 2H, NCH$_2$) ppm; **$^{13}$C{$^1$H} NMR (151 MHz, toluene-*d*8, 95 °C)**: $\delta$ = 160.9, 136.7, 132.6, 129.1, 128.2, 128.0, 118.5, 94.3, 51.7, 51.1 ppm; **IR (ATR)**: $\nu$ = 3346 (w), 3076 (w), 3031 (w), 2931 (w), 2669 (br w), 2329 (br w), 2088 (br w), 1877 (br w), 1676 (s), 1496 (w), 1414 (s), 1355 (w), 1284 (w), 1226 (m), 1165 (w), 1122 (w), 1077 (w), 1029 (w), 992 (w), 934 (m), 810 (s), 738 (m), 698 (m), 668 (m) cm$^{-1}$; **MS (100 eV, CI)**: *m*/*z* (%): 292 (69) [*M*+H]$^+$; **MS (70 eV, EI)**: *m*/*z* (%): 291 (12) [*M*]$^+$, 256 (76), 250 (11), 221 (10), 117 (11), 91 (100) [$C_7H_7$]$^+$.

**Analytical data of ATRC products 2**

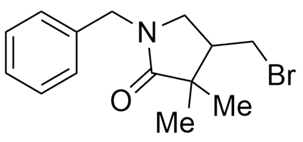

1-Benzyl-4-(bromomethyl)-3,3-dimethyl-2-pyrrolidinone (**2a**): The title compound was synthesized using general procedure GP3 and was obtained as a colorless oil (0.0848 g, 0.29 mmol, 84%) after column chromatography ($SiO_2$/*n*-Pentane:EtOAc 3:1 *v/v*). $\boldsymbol{R_f}$ = 0.23 (*n*-Pentane:EtOAc 3:1 *v/v*), slightly UV-active, stains with $KMnO_4$; **$^1$H NMR (600 MHz, $CDCl_3$)**: $\delta$ = 7.32 (m, 2H, Ar–*H*), 7.27 (m, 1H, Ar–*H*), 7.20 (m, 2H, Ar–*H*), 4.52 (d, $^3J_{H,H}$ = 14.6 Hz, 1H, PhC$H_AH_B$N), 4.37 (d, $^3J_{H,H}$ = 14.6 Hz, 1H, PhC$H_AH_B$N), 3.47 (dd, $^3J_{H,H}$ = 10.1, 4.8 Hz, 1H, C$H_AH_B$Br), 3.37 (dd, $^3J_{H,H}$ = 10.1, 7.6 Hz, 1H, C$H_AH_B$Br), 3.23 (t, $^3J_{H,H}$ = 10.4 Hz, 1H, NC$H_AH_B$), 2.89 (dd, $^3J_{H,H}$ = 10.1, 8.6 Hz, NC$H_AH_B$), 2.42 (dddd, $^3J_{H,H}$ = 10.8, 8.7, 7.6, 4.8 Hz, 1H, C*H*), 1.24 (s, 3H, (C=O)C($CH_3$)$CH_3$), 1.00 (s, 3H, (C=O)C$CH_3$($CH_3$)) ppm; **$^{13}$C{$^1$H} NMR (151 MHz, $CDCl_3$)**: $\delta$ = 178.5, 136.4, 128.9, 128.1, 127.8, 49.0, 46.8, 46.1, 44.1, 31.5, 24.3, 18.4 ppm; **IR (ATR)**: $\nu$ = 3030 (w), 2965 (w), 2927 (w), 2867 (w), 2320 (br w), 2162 (w), 2059 (w), 1685 (s), 1605 (w), 1491 (m), 1433 (s), 1360 (m), 1321 (w), 1269 (m), 1165 (w), 1080 (w), 1034 (w), 998 (w), 947 (w), 810 (w), 747 (m), 700 (m), 673 (m) $cm^{-1}$; **MS (100 eV, CI)**: *m*/*z* (%): 296 (98) $[M+H]^+$; **MS (70 eV, EI)**: *m*/*z* (%): 296 (47) $[M+H]^+$, 295 (49) $[M]^+$, 280 (22), 216 (10), 204 (12), 106 (19), 91 (100) $[C_7H_7]^+$, 65 (20). The NMR data closely match the ones previously reported in the literature.[5]

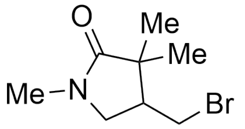

4-(Bromomethyl)-1,3,3-trimethyl-2-pyrrolidinone (**2b**): The title compound was synthesized using general procedure GP3 and was obtained as a colorless, viscous oil (0.0545 g, 0.25 mmol, 73%) after column chromatography ($SiO_2$/*n*-Pentane:EtOAc 1:1 *v/v*). $\boldsymbol{R_f}$ = 0.15 (*n*-Pentane:EtOAc 1:1 *v/v*), stains with $KMnO_4$; **$^1$H NMR (600 MHz, $CDCl_3$)**: $\delta$ = 3.49 (m, 2H, NC$H_2$), 3.29 (t, $^3J_{H,H}$ = 10.4 Hz, 1H, C$H_AH_B$Br), 3.05 (dd, $^3J_{H,H}$ = 10.0, 8.4 Hz, C$H_AH_B$Br), 2.84 (s, 3H, N$CH_3$), 2.44 (m, 1H, CH), 1.19 (s, 3H, (C=O)C($CH_3$)$CH_3$), 0.97 (s, 3H, (C=O)C$CH_3$($CH_3$)) ppm; **$^{13}$C{$^1$H} NMR (151 MHz, $CDCl_3$)**: $\delta$ = 178.6, 51.8, 46.2, 43.9, 31.7, 29.9, 24.5, 18.5 ppm; **IR (ATR)**: $\nu$ = 3860 (br w), 3473 (br w), 2964 (m), 2927 (m), 2870 (w), 2670 (w), 2320 (br w), 2095 (br w), 1991 (w), 1906 (br w), 1682 (s), 1496 (m), 1438 (m), 1401 (m), 1362 (m), 1274 (m), 1173 (w), 1083 (m), 1024 (w), 971 (w), 845 (w), 807 (w), 753 (w), 673 (m) $cm^{-1}$; **MS (100 eV, CI)**: *m*/*z* (%): 220 (61) $[M+H]^+$; **MS (70 eV, EI)**: *m*/*z* (%): 220 (36) $[M+H]^+$, 219 (32) $[M]^+$, 140 (100), 126 (44); **HRMS (ESI)**: *m*/*z* calculated for $C_8H_{14}BrNO+Na^+$: 242.0151 $[M+Na]^+$; found: 242.0149.

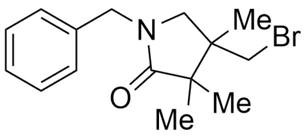

1-Benzyl-4-(1-bromomethyl)-3,3,4-trimethyl-2-pyrrolidinone (**2c**): The title compound was synthesized using general procedure GP3 and was obtained as a colorless oil (0.0232 g, 0.07 mmol, 22%; 180 min of milling: 0.0742 g, 0.24 mmol, 71%) after column chromatography ($SiO_2$/*n*-Pentane:EtOAc 3:1 *v/v*). $\boldsymbol{R_f}$ =0.23 (*n*-Pentane:EtOAc 3:1 *v/v*), slightly UV-active, stains with $KMnO_4$; **$^1$H NMR (600 MHz, $CDCl_3$)**: $\delta$ = 7.33 (m, 2H, Ar–*H*), 7.28 (m, 1H, Ar–*H*), 7.21 (m, 2H, Ar–*H*), 4.45 (m, 2H, PhC$H_2$N), 3.42 (d, $^3J_{H,H}$ = 10.2 Hz, 1H, C$H_AH_B$Br), 3.34 (d, $^3J_{H,H}$ = 10.2 Hz, 1H, C$H_AH_B$Br), 3.18 (d, $^3J_{H,H}$ = 10.2 Hz, 1H, NC$H_AH_B$), 2.83 (d, $^3J_{H,H}$ = 10.2 Hz, 1H, NC$H_AH_B$), 1.12 (s, 3H, (C=O)C($CH_3$)$CH_3$), 1.10 (s, 3H, $CH_3$), 1.07 (s, 3H, (C=O)C$CH_3$($CH_3$)) ppm; **$^{13}$C{$^1$H} NMR (151 MHz, $CDCl_3$)**: $\delta$ = 178.5, 136.5, 128.9, 128.3, 127.8, 54.4, 47.2, 46.8, 43.0, 40.5, 20.3, 20.0, 19.3 ppm; **IR (ATR)**: $\nu$ = 3490 (br w), 3030 (w), 2972 (m), 2928 (w), 2872 (w), 2161 (w), 1970 (w), 1685 (s), 1606 (w), 1493 (m), 1432 (s), 1376 (m), 1310 (m), 1263 (m), 1165 (m), 1111 (w), 1079 (w), 1028 (w), 950 (w), 853 (w), 815 (w), 748 (m), 700 (s), 668 (m) $cm^{-1}$; **MS (100 eV, CI)**: *m*/*z* (%): 310 (100) $[M+H]^+$; **MS (70 eV, EI)**: *m*/*z* (%): 310 (19) $[M+H]^+$, 309 (45) $[M]^+$, 294 (23), 230 (16), 91 (100) $[C_7H_7]^+$, 65 (17); **HRMS (ESI)**: *m*/*z* calculated for $C_{15}H_{20}BrNO+Na^+$: 332.0621 $[M+Na]^+$; found: 332.0620.

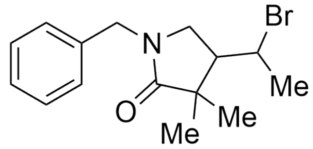

1-Benzyl-4-(1-bromoethyl)-3,3-dimethyl-2-pyrrolidinone (**2d**): The title compound was synthesized using general procedure GP3 and was obtained as a colorless solid mixture of diastereomers (dr = 64:36) (0.0611 g, 0.20 mmol, 58%; 180 min of milling: 0.0889 g, 0.29 mmol, 85%) after column chromatography ($SiO_2$/*n*-Pentane:EtOAc 4:1 *v/v*). ***R*f** = 0.22 (*n*-Pentane:EtOAc 4:1 *v/v*), slightly UV-active, stains with $KMnO_4$; **m.p.**: 64.7-68.0 °C; **$^1$H NMR (600 MHz, $CDCl_3$)**: $\delta$ (minor diastereomer) = 7.33 (m, 2H, Ar–*H*), 7.28 (m, 1H, Ar–*H*), 7.21 (m, 2H, Ar–*H*), 4.51 (t, $^3J_{H,H}$ = 14.3 Hz, 1H, PhC$H_A$H$_B$N), 4.39 (dd, $^3J_{H,H}$ = 14.6, 11.1 Hz, 1H, PhCH$_A$$H_B$N), 4.08 (dq, $^3J_{H,H}$ = 9.4, 6.6 Hz, 1H, $CH_3$C*H*Br), 3.23 (dd, $^3J_{H,H}$ = 9.9, 8.0 Hz, 1H, NC$H_A$H$_B$), 2.81 (dd, $^3J_{H,H}$ = 10.0, 8.7 Hz, 1H, NCH$_A$$H_B$), 2.43 (q, $^3J_{H,H}$ = 8.7 Hz, 1H, $CH_2$C*H*C$(CH_3)_2$), 1.59 (d, $^3J_{H,H}$ = 6.6 Hz, 3H, CHC$H_3$), 1.41 (s, 3H, (C=O)C(C$H_3$)$CH_3$), 1.13 (s, 3H, (C=O)C$CH_3$(C$H_3$)) ppm; $\delta$ (major diastereomer) = 7.33 (m, 2H, Ar–*H*), 7.28 (m, 1H, Ar–*H*), 7.21 (m, 2H, Ar–*H*), 4.51 (t, $^3J_{H,H}$ = 14.3 Hz, 1H, PhC$H_A$H$_B$N), 4.39 (dd, $^3J_{H,H}$ = 14.6, 11.1 Hz, 1H, PhCH$_A$$H_B$N), 4.14 (dq, $^3J_{H,H}$ = 10.2, 6.6 Hz, 1H, $CH_3$C*H*Br), 3.38 (dd, $^3J_{H,H}$ = 10.3, 7.8 Hz, 1H, NC$H_A$H$_B$), 2.92 (t, $^3J_{H,H}$ = 9.9 Hz, 1H, NCH$_A$$H_B$), 2.29 (td, $^3J_{H,H}$ = 9.8, 7.7 Hz, 1H, $CH_2$C*H*C$(CH_3)_2$), 1.82 (d, $^3J_{H,H}$ = 6.6 Hz, 3H, CHC$H_3$), 1.32 (s, 3H, (C=O)C(C$H_3$)$CH_3$), 1.03 (s, 3H, (C=O)C$CH_3$(C$H_3$))) ppm; **$^{13}$C{$^1$H} NMR (151 MHz, $CDCl_3$)**: $\delta$ (minor diastereomer) = 179.1, 136.4, 128.9, 128.2, 127.9, 51.7, 47.5, 47.4, 46.7, 44.2, 25.6, 24.4, 18.0 ppm; $\delta$ (major diastereomer) = 178.7, 136.5, 128.9, 128.2, 127.8, 52.3, 51.0, 50.2, 46.8, 44.3, 26.2, 25.6, 18.0 ppm; **IR (ATR)**: $\nu$ = 3348 (w), 3032 (w), 2969 (m), 2930 (m), 2869 (w), 2163 (w), 1960 (w), 1805 (w), 1678 (s), 1491 (m), 1436 (s), 1362 (m), 1319 (m), 1293 (m), 1261 (s), 1177 (m), 1120 (w), 1078 (w), 1030 (m), 1009 (m), 954 (m), 932 (m), 810 (w), 740 (s), 697 (s), 658 (w) $cm^{-1}$; **MS (100 eV, CI)**: *m*/*z* (%): 310 (96) $[M+H]^+$; **MS (70 eV, EI)**: *m*/*z* (%): 310 (37) $[M+H]^+$, 309 (58) $[M]^+$, 294 (25), 230 (16), 202 (19), 91 (100) $[C_7H_7]^+$, 65 (17); **HRMS (ESI)**: *m*/*z* calculated for $C_{15}H_{20}BrNO+Na^+$: 332.0621 $[M+Na]^+$; found: 332.0623.

**References**

[1] Mestrelab Research S.L., MestReNova, Version 12.0.1-20560, **2018**.

[2] G. R. Fulmer, A. J. M. Miller, N. H. Sherden, H. E. Gottlieb, A. Nudelman, B. M. Stoltz, J. E. Bercaw, K. I. Goldberg, *Organometallics* **2019**, *29*, 2176–2179.

[3] J. B. Sweeney, A. K. Ball, P. A. Lawrence, M. C. Sinclair, L. J. Smith, *Angew. Chem. Int. Ed.* **2018**, *57*, 10202-10206; *Angew. Chem.* **2018**, *130*, 10359–10363.

[4] D. Paripovic, H.-A. Klok, *Macromol. Chem. Phys.* **2011**, *212*, 950–958.

[5] A. J. Clark, A. E. C. Collis, D. J. Fox, L. L. Halliwell, N. James, R. K. O'Reilly, H. Parekh, A. Ross, A. B. Sellars, H. Willcock, P. Wilson, *J. Org. Chem.* **2012**, *77*, 6778–6788.

**[1]H and [13]C{[1]H} NMR spectra of new starting materials and ATRC products**

[1]H NMR spectrum (600 MHz, toluene-$d_8$, 95 °C) of *N*-allyl-2-bromo-*N*-2-dimethylpropanamide (**1b**)

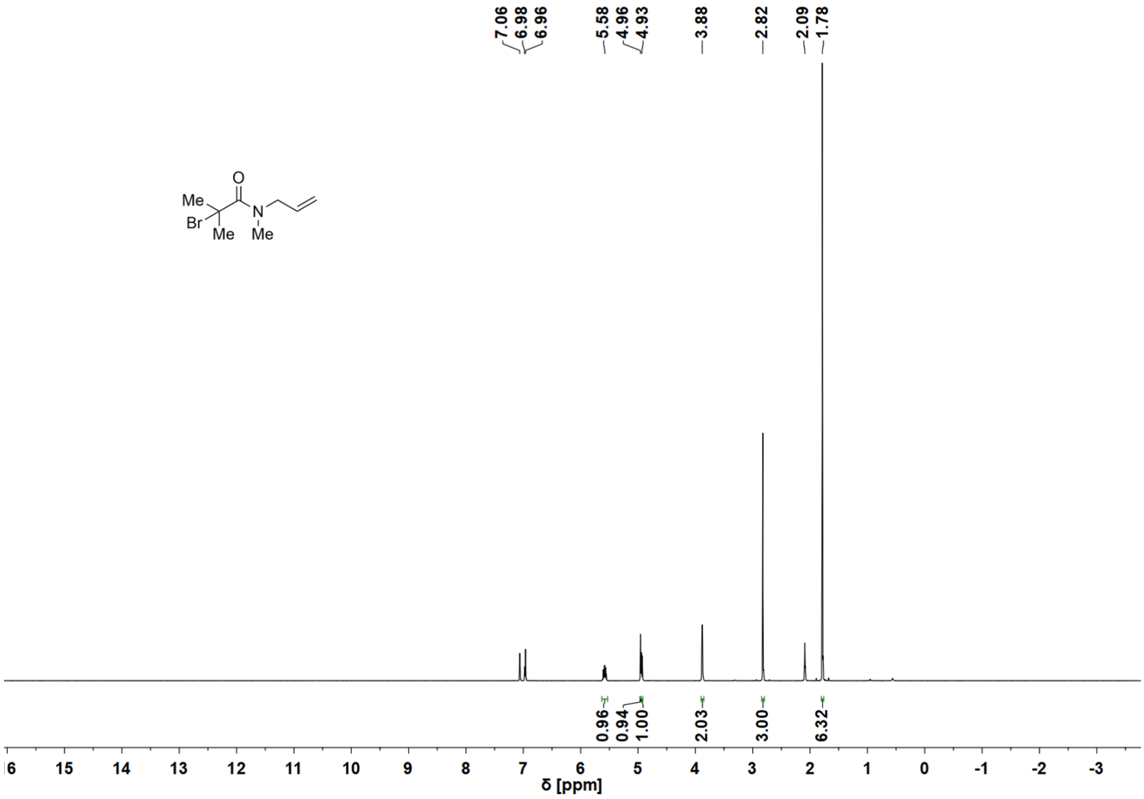


[13]C{[1]H} NMR spectrum (101 MHz, toluene-$d_8$, 95 °C) of *N*-allyl-2-bromo-*N*-2-dimethylpropanamide (**1b**)

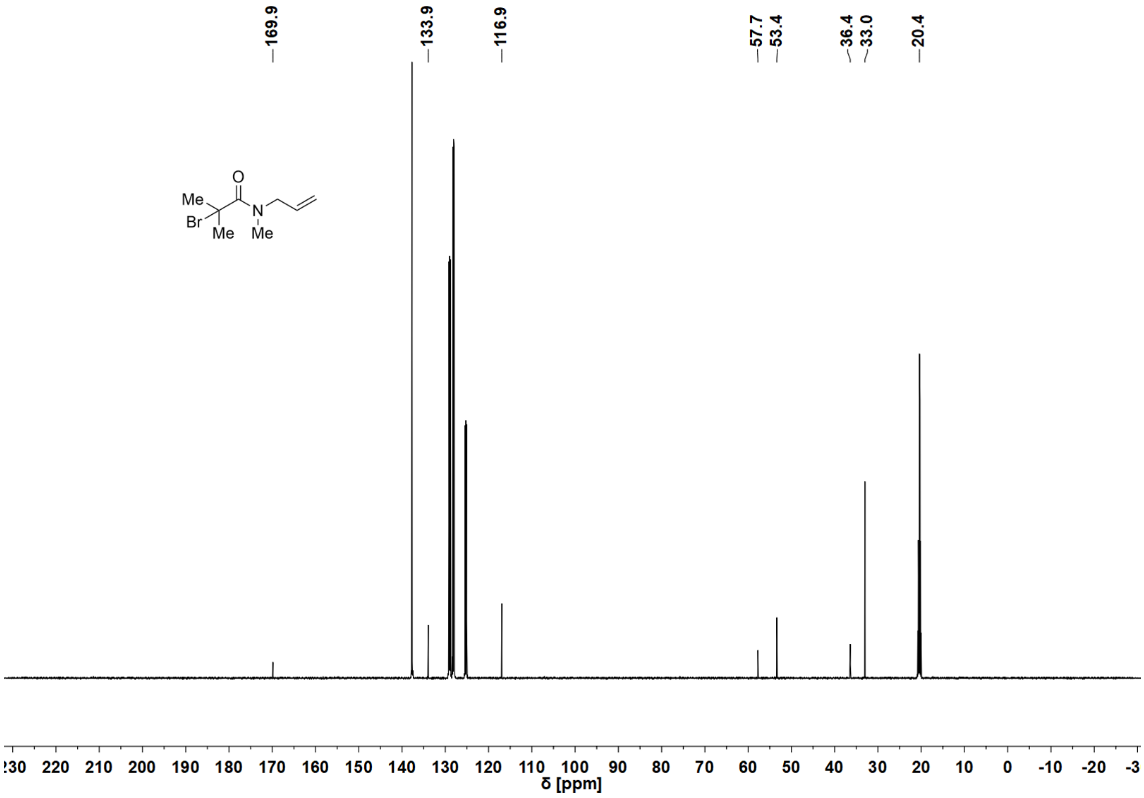

$^{1}$H NMR spectrum (600 MHz, toluene-$d_8$, 95 °C) of *N*-benzyl-2-bromo-2-methyl-*N*-(2-methylallyl)propanamide (**1c**)

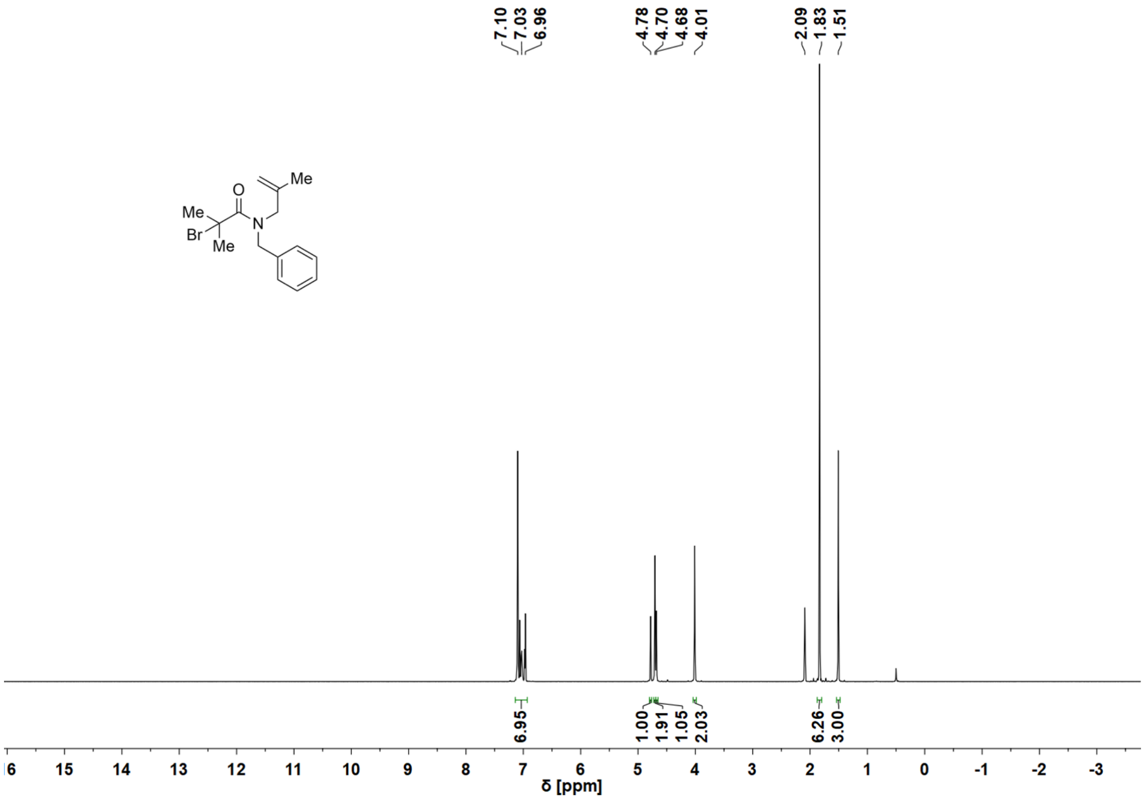


$^{13}$C{$^{1}$H} NMR spectrum (151 MHz, toluene-$d_8$, 95 °C) of *N*-benzyl-2-bromo-2-methyl-*N*-(2-methylallyl)propanamide (**1c**)

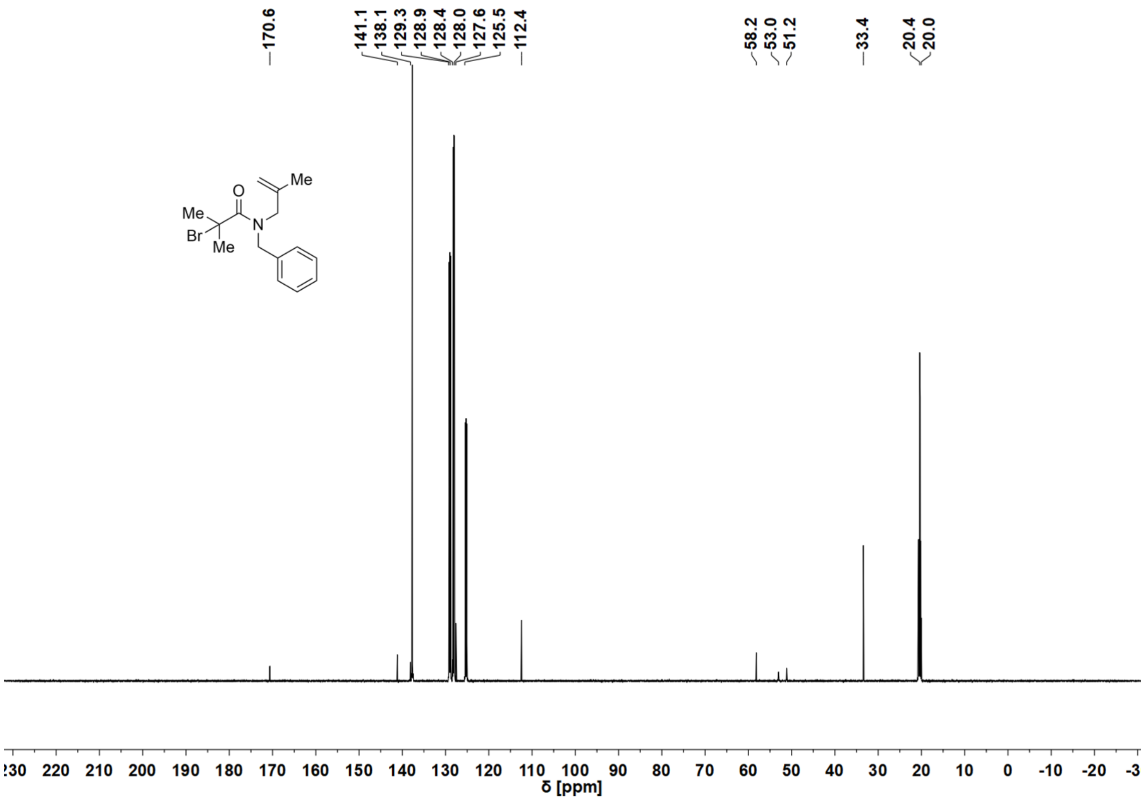

$^1$H NMR spectrum (400 MHz, toluene-$d_8$, 95 °C) of *N*-benzyl-2-bromo-*N*-crotyl-2-methylpropanamide (**1d**)

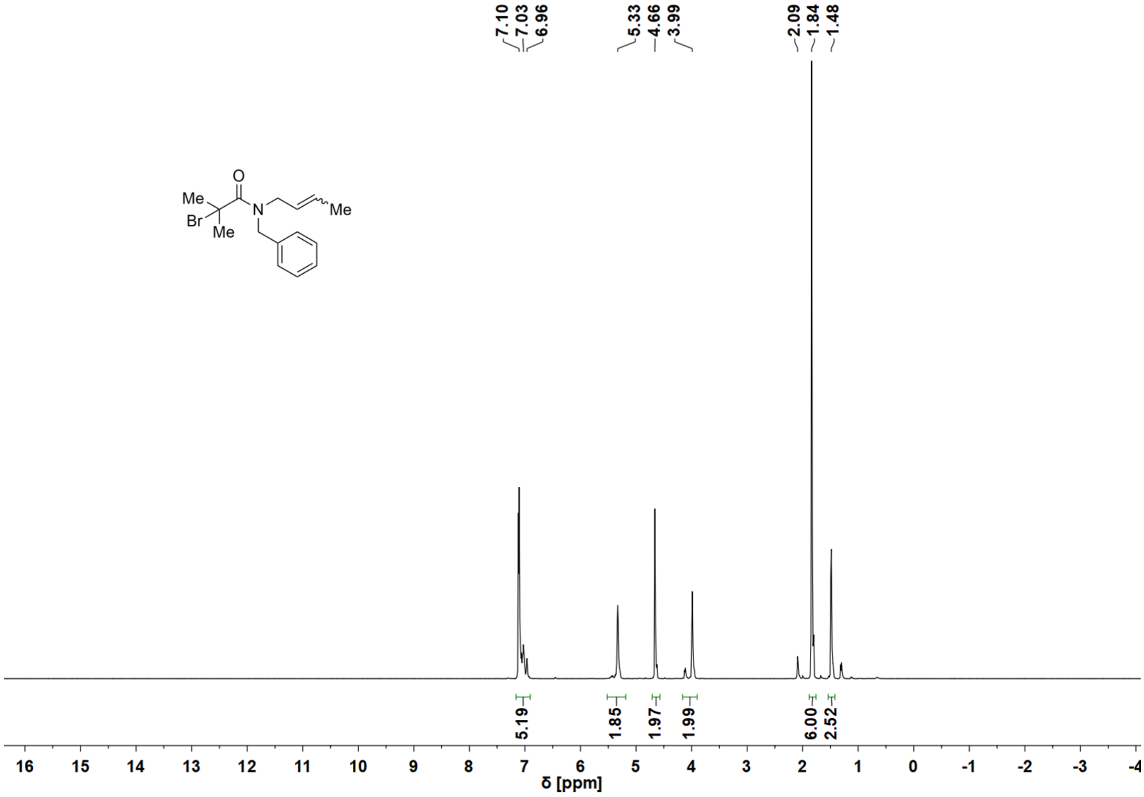


$^{13}$C{$^1$H} NMR spectrum (101 MHz, toluene-$d_8$, 95 °C) of *N*-benzyl-2-bromo-*N*-crotyl-2-methylpropanamide (**1d**)

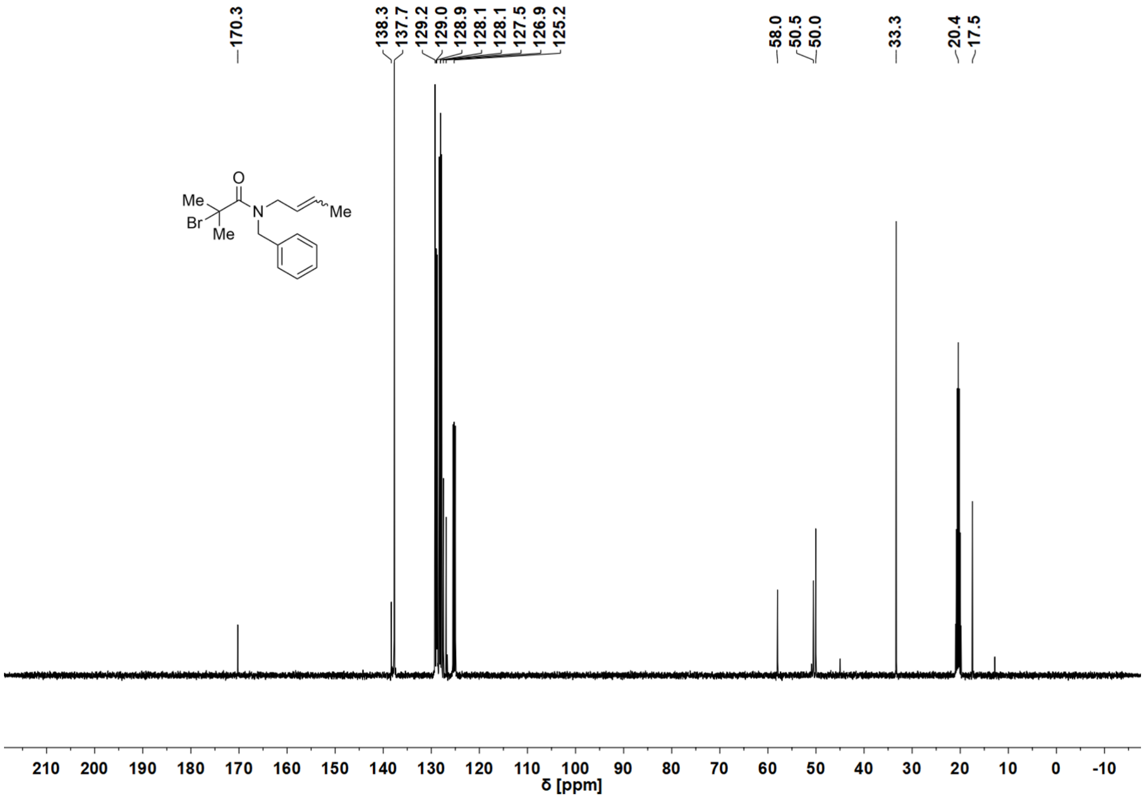

[1]H NMR spectrum (600 MHz, $CDCl_3$) of 1-benzyl-4-(bromomethyl)-3,3-dimethyl-2-pyrrolidinone (**2a**)

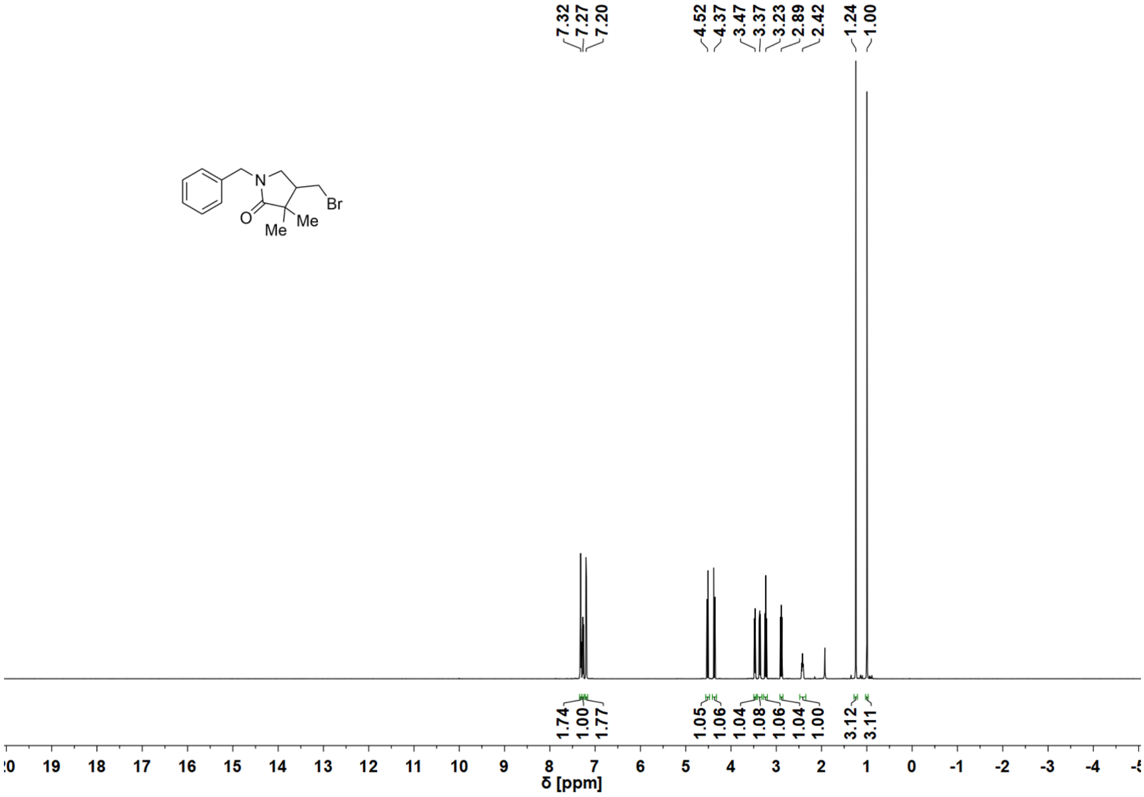


[13]C{[1]H} NMR spectrum (151 MHz, $CDCl_3$) of 1-benzyl-4-(bromomethyl)-3,3-dimethyl-2-pyrrolidinone (**2a**)

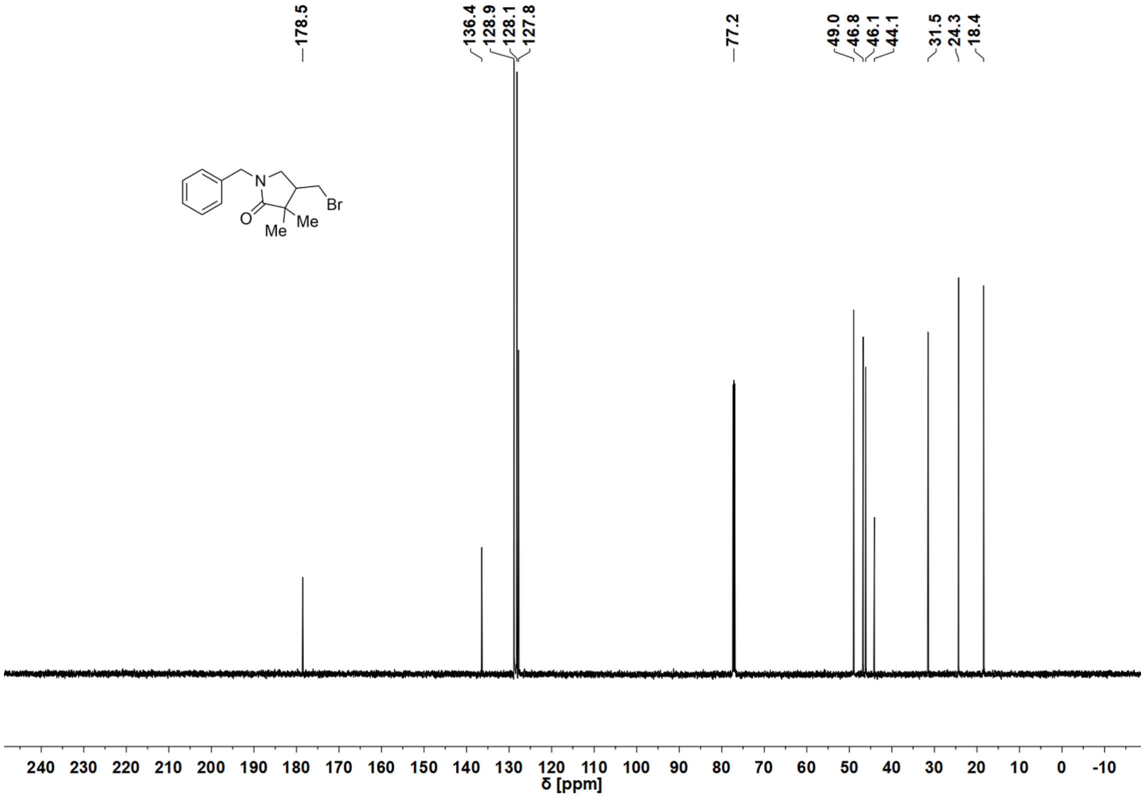

$^1$H NMR spectrum (600 MHz, $CDCl_3$) of 4-(bromomethyl)-1,3,3-trimethyl-2-pyrrolidinone (**2b**)

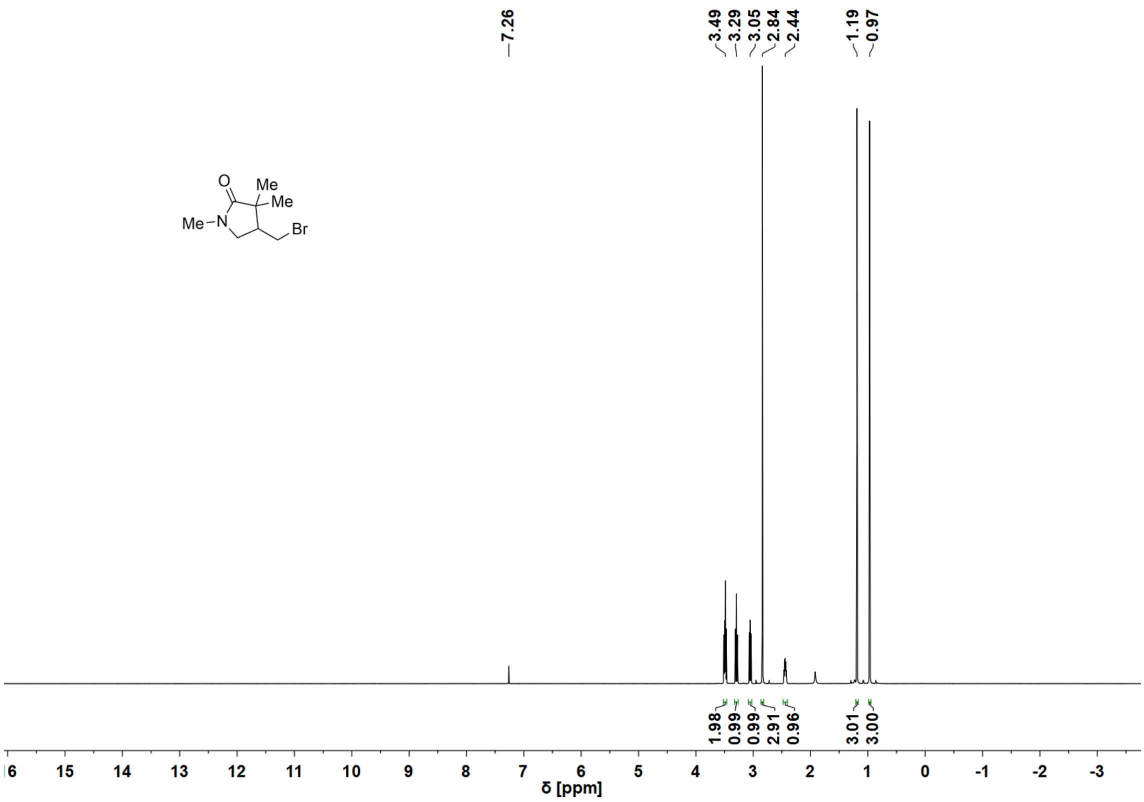


$^{13}C\{^1H\}$ NMR spectrum (151 MHz, $CDCl_3$) of 4-(bromomethyl)-1,3,3-trimethyl-2-pyrrolidinone (**2b**)

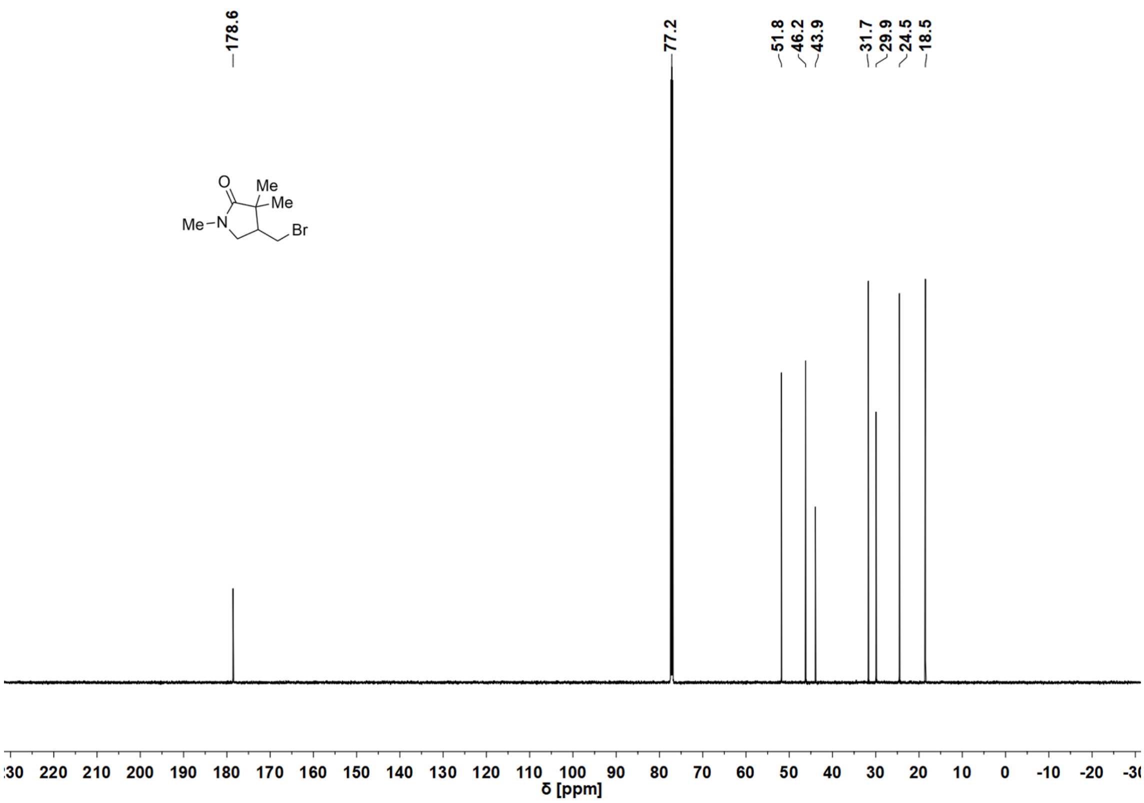

[1]H,[1]H COSY spectrum ($CDCl_3$) of 4-(bromomethyl)-1,3,3-trimethyl-2-pyrrolidinone (**2b**)

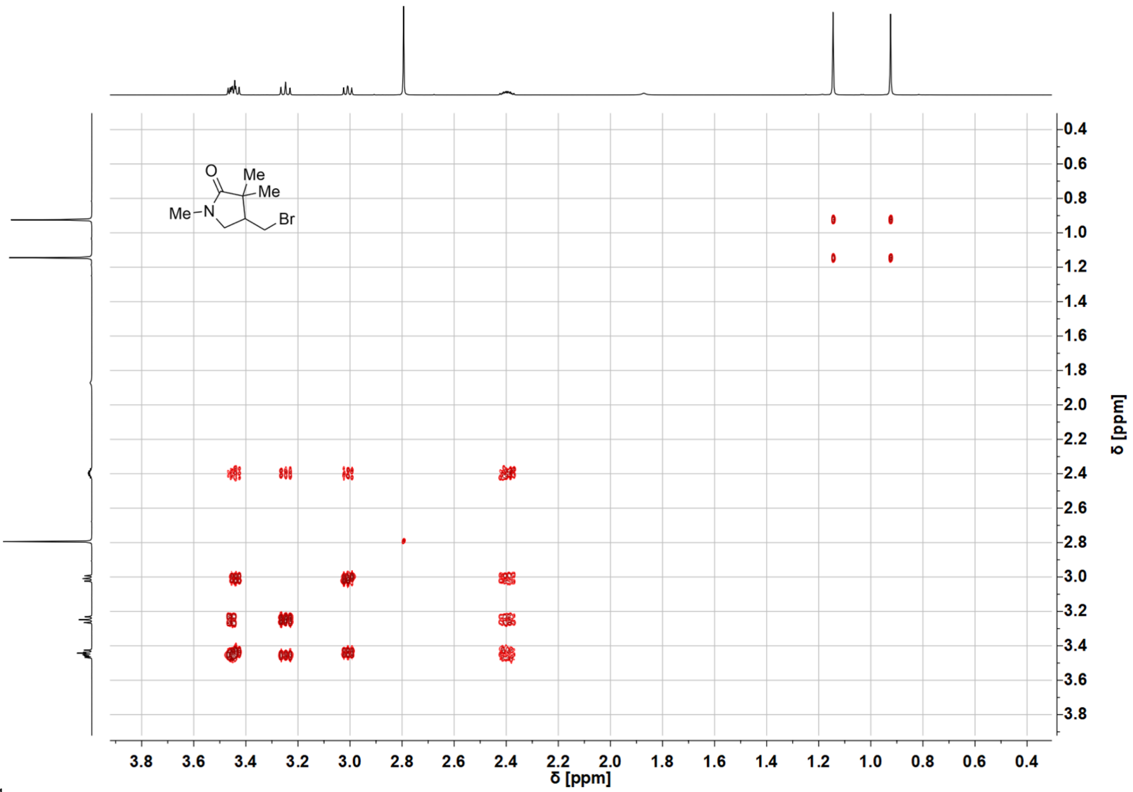


[1]H,[13]C{[1]H} HSQC spectrum ($CDCl_3$) of 4-(bromomethyl)-1,3,3-trimethyl-2-pyrrolidinone (**2b**)

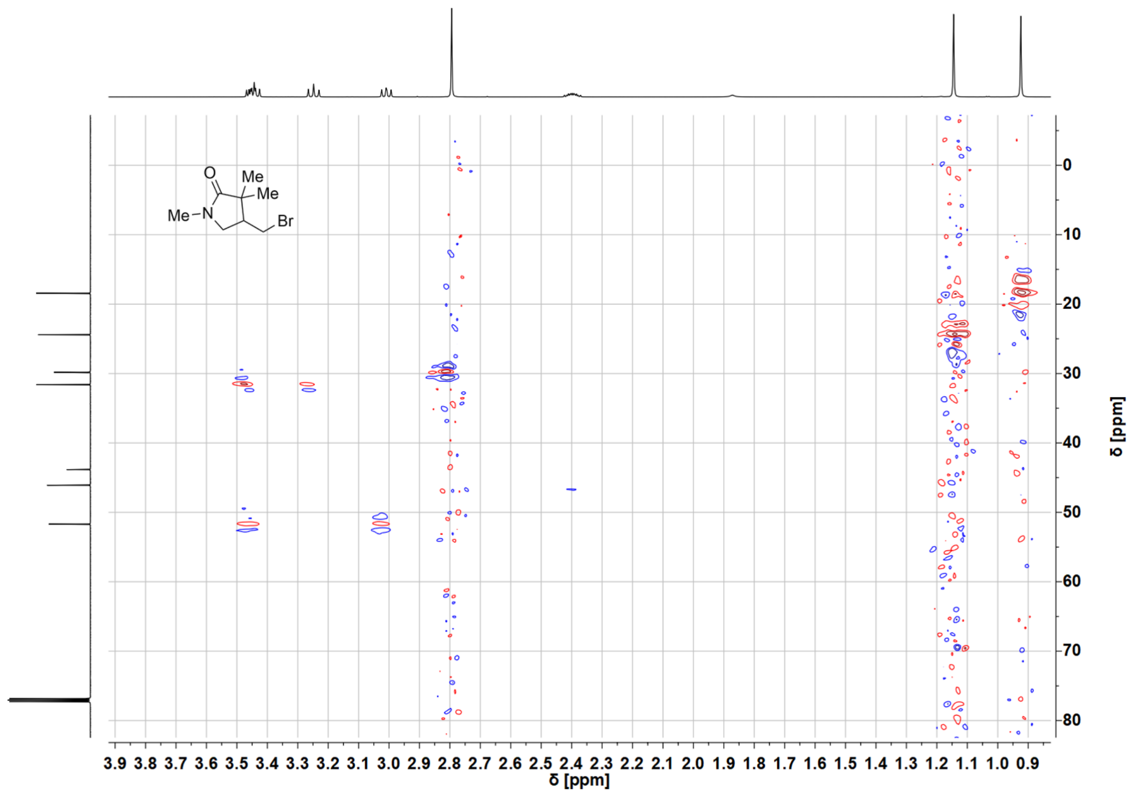

[1]H,[13]C{[1]H} HMBC spectrum ($CDCl_3$) of 4-(bromomethyl)-1,3,3-trimethyl-2-pyrrolidinone (**2b**)

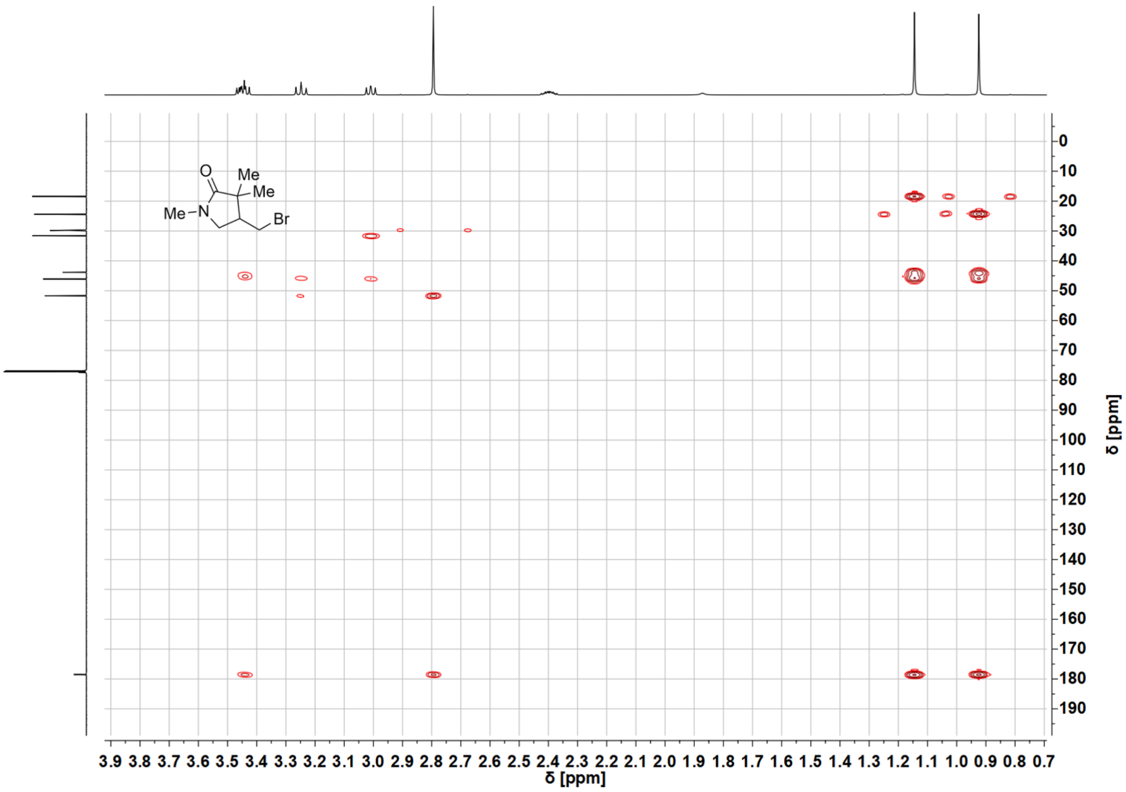

[1]H NMR spectrum (600 MHz, $CDCl_3$) of 1-benzyl-4-(1-bromomethyl)-3,3,4-trimethyl-2-pyrrolidinone (**2c**)

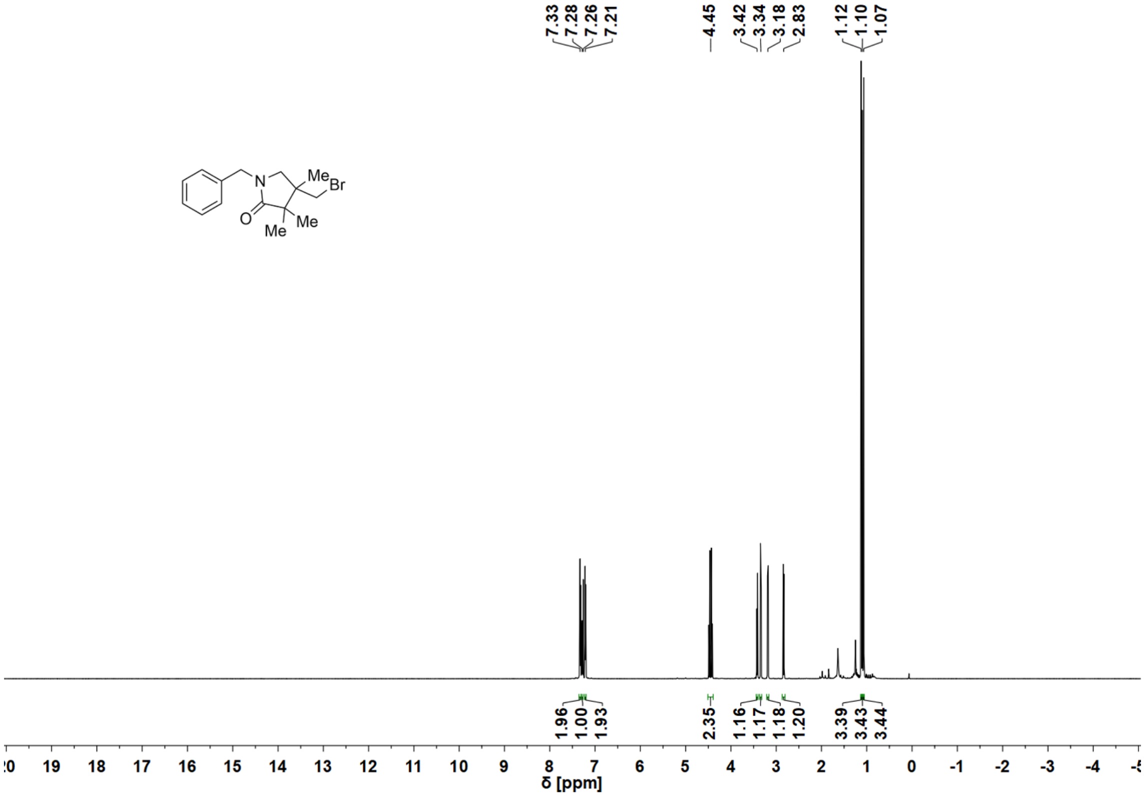

$^{13}C\{^1H\}$ NMR spectrum (151 MHz, $CDCl_3$) of 1-benzyl-4-(1-bromomethyl)-3,3,4-trimethyl-2-pyrrolidinone (**2c**)

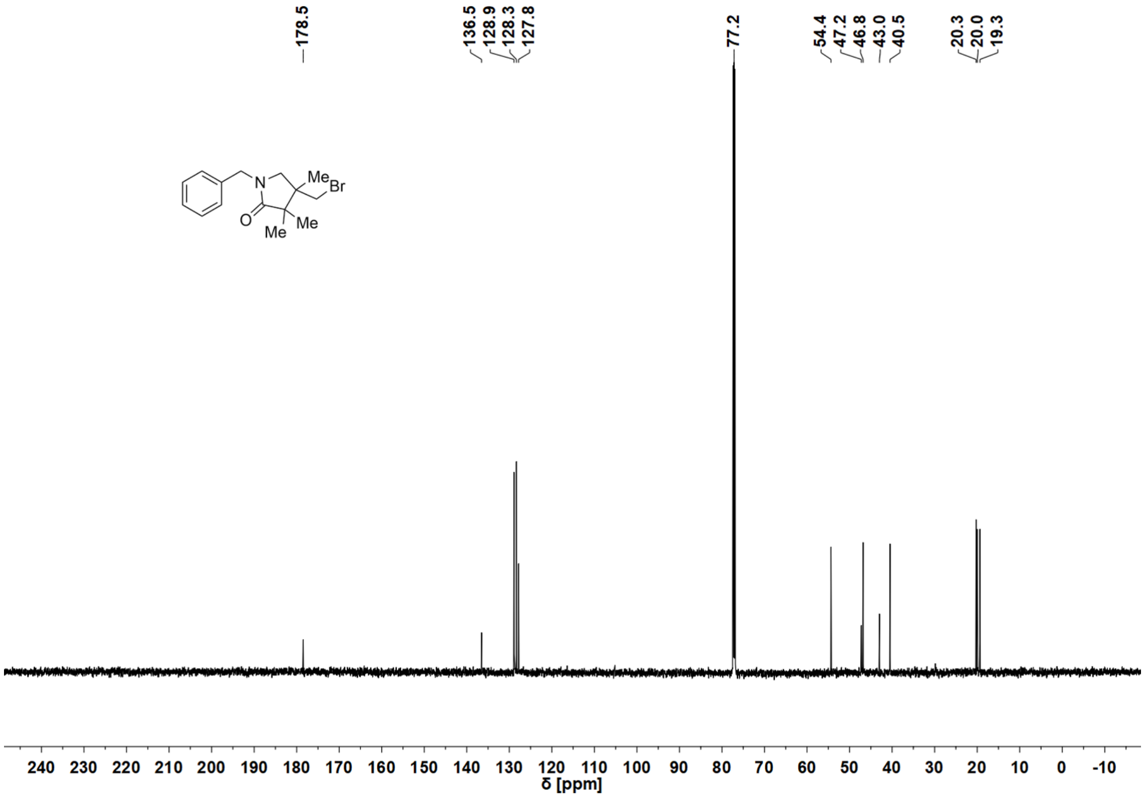


$^1H,^1H$ COSY spectrum ($CDCl_3$) of 1-benzyl-4-(1-bromomethyl)-3,3,4-trimethyl-2-pyrrolidinone (**2c**)

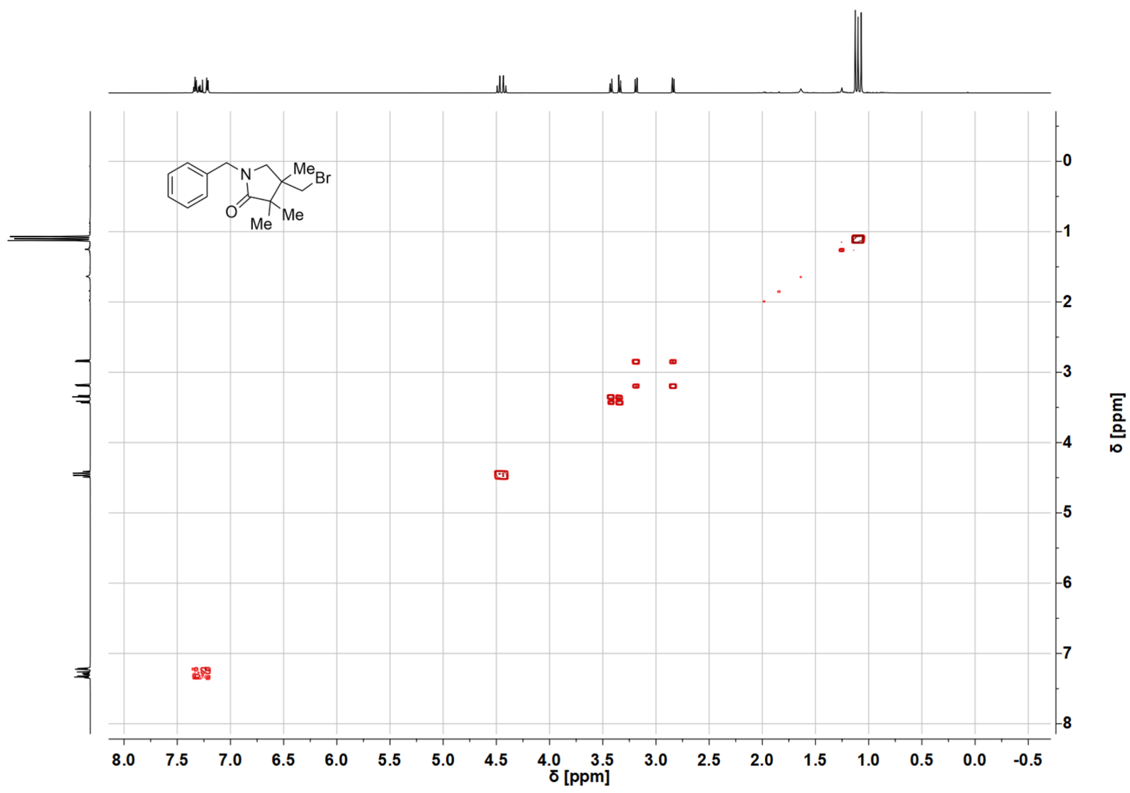

$^{1}H,^{13}C\{^{1}H\}$ HSQC spectrum ($CDCl_3$) of 1-benzyl-4-(1-bromomethyl)-3,3,4-trimethyl-2-pyrrolidinone (**2c**)

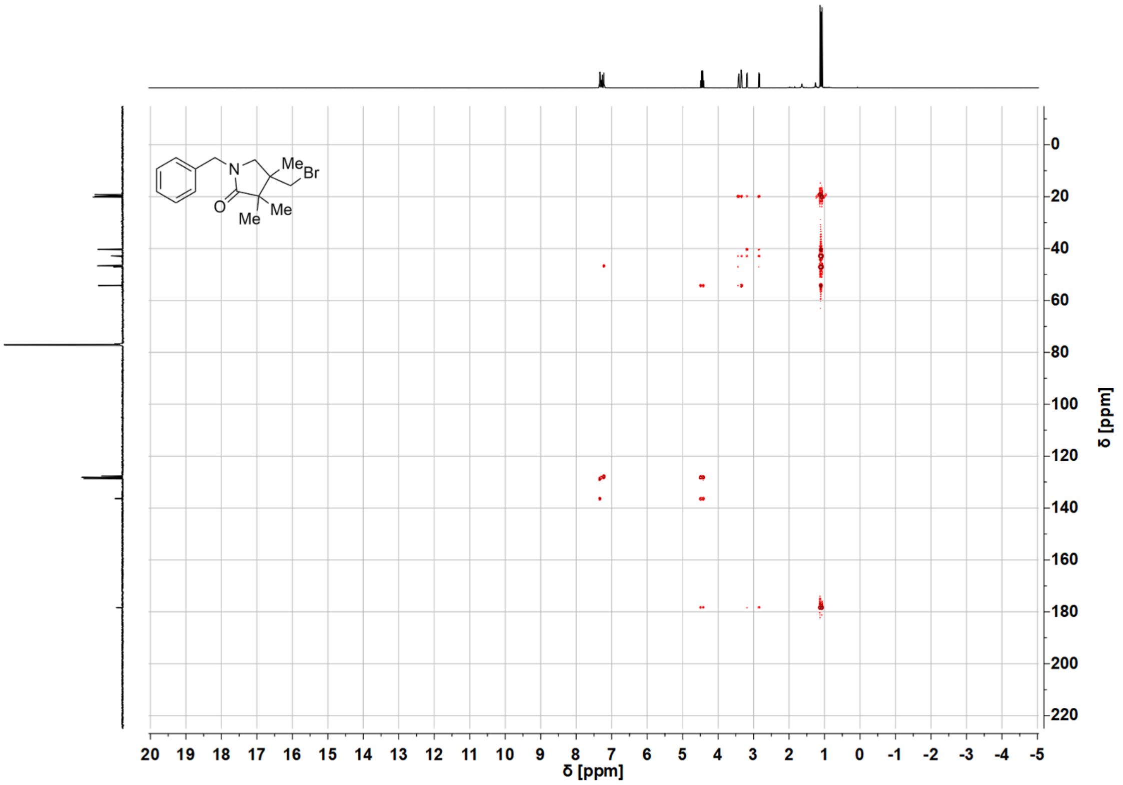


$^{1}H,^{13}C\{^{1}H\}$ HMBC spectrum ($CDCl_3$) of 1-benzyl-4-(1-bromomethyl)-3,3,4-trimethyl-2-pyrrolidinone (**2c**)

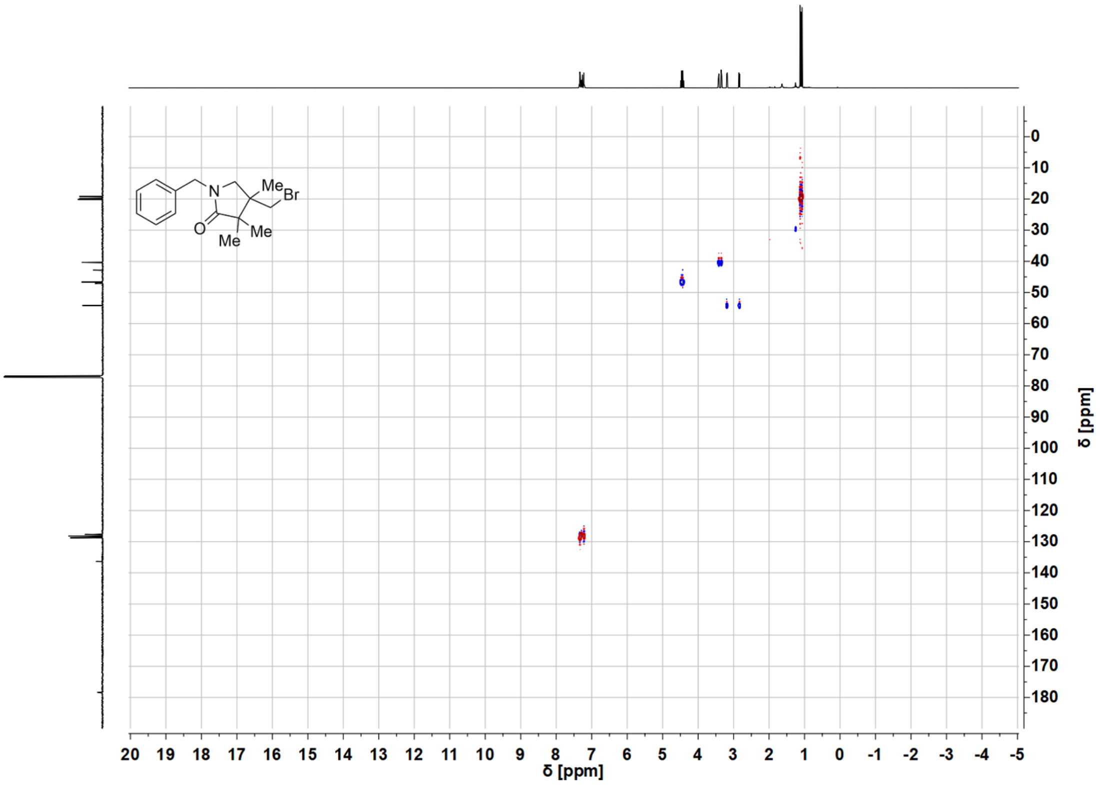

[1]H NMR spectrum (600 MHz, $CDCl_3$) of 1-benzyl-4-(1-bromoethyl)-3,3-dimethyl-2-pyrrolidinone (**2d**)

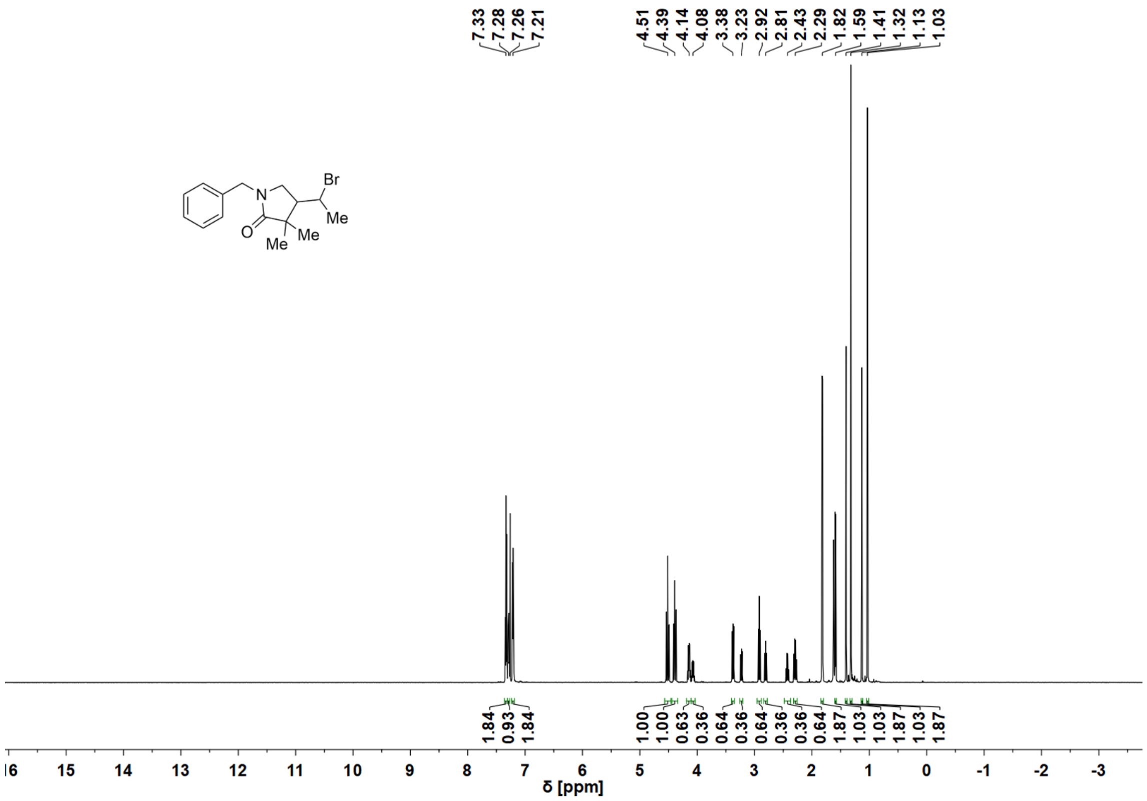

[13]C{[1]H} NMR spectrum (151 MHz, $CDCl_3$) of 1-benzyl-4-(1-bromoethyl)-3,3-dimethyl-2-pyrrolidinone (**2d**)

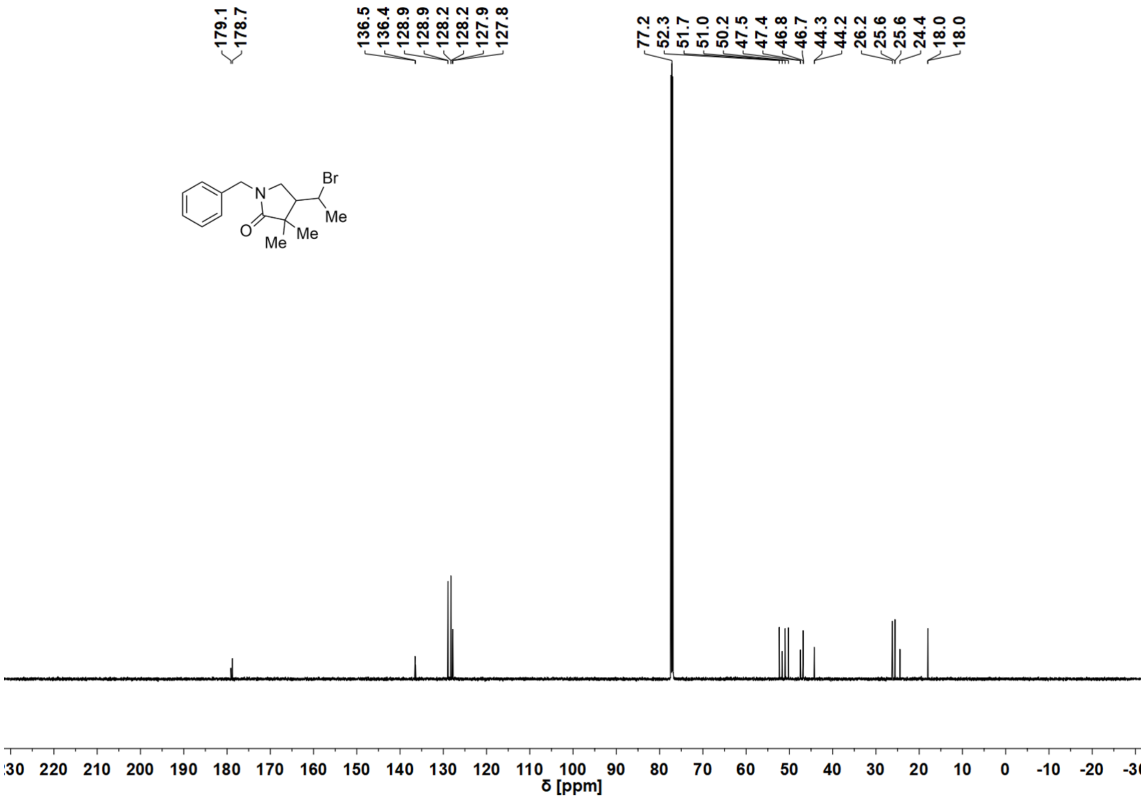

$^{1}H,^{1}H$ COSY spectrum ($CDCl_3$) of 1-benzyl-4-(1-bromoethyl)-3,3-dimethyl-2-pyrrolidinone (**2d**)

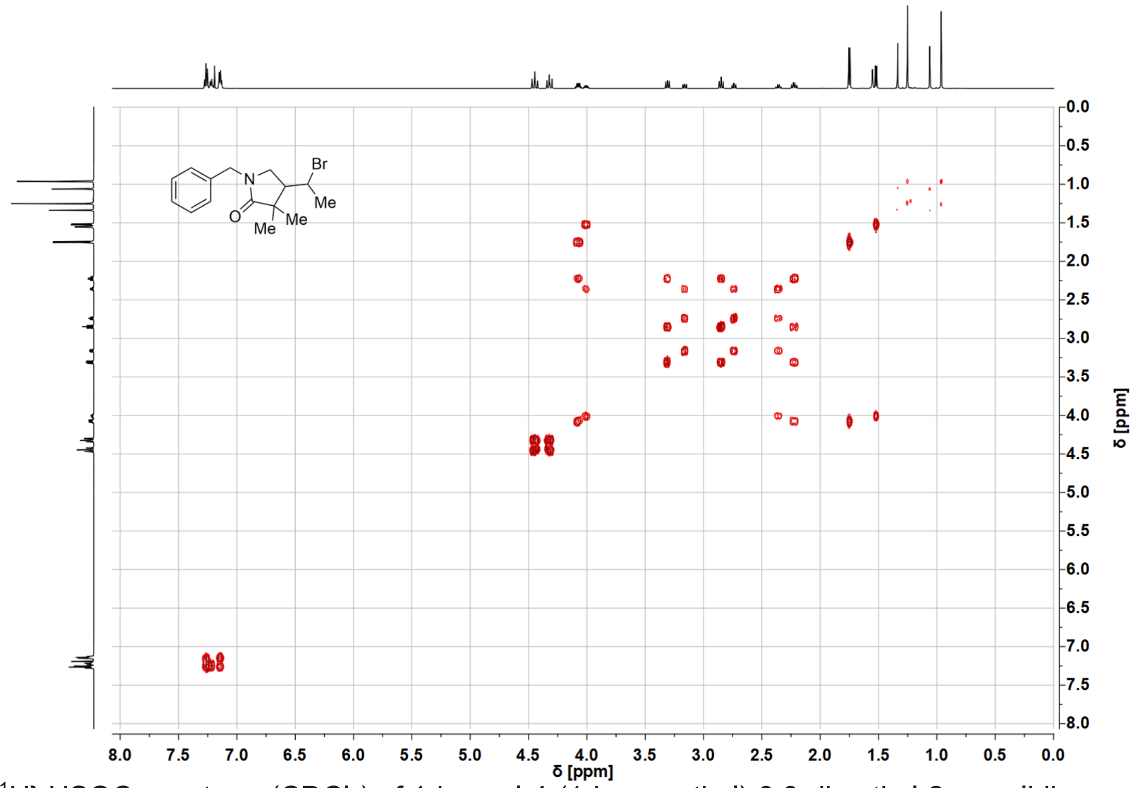


$^{1}H,^{13}C\{^{1}H\}$ HSQC spectrum ($CDCl_3$) of 1-benzyl-4-(1-bromoethyl)-3,3-dimethyl-2-pyrrolidinone (**2d**)

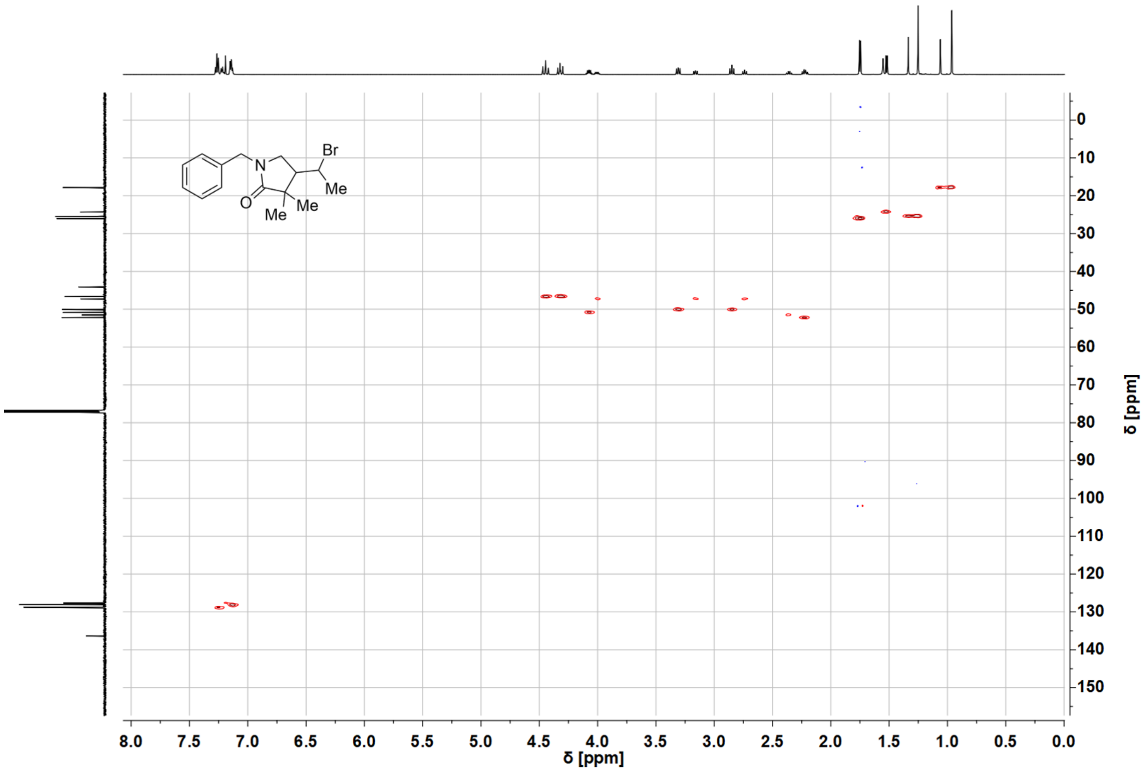

$^{1}H$,$^{13}C\{^{1}H\}$ HMBC spectrum ($CDCl_3$) of 1-benzyl-4-(1-bromoethyl)-3,3-dimethyl-2-pyrrolidinone (**2d**)

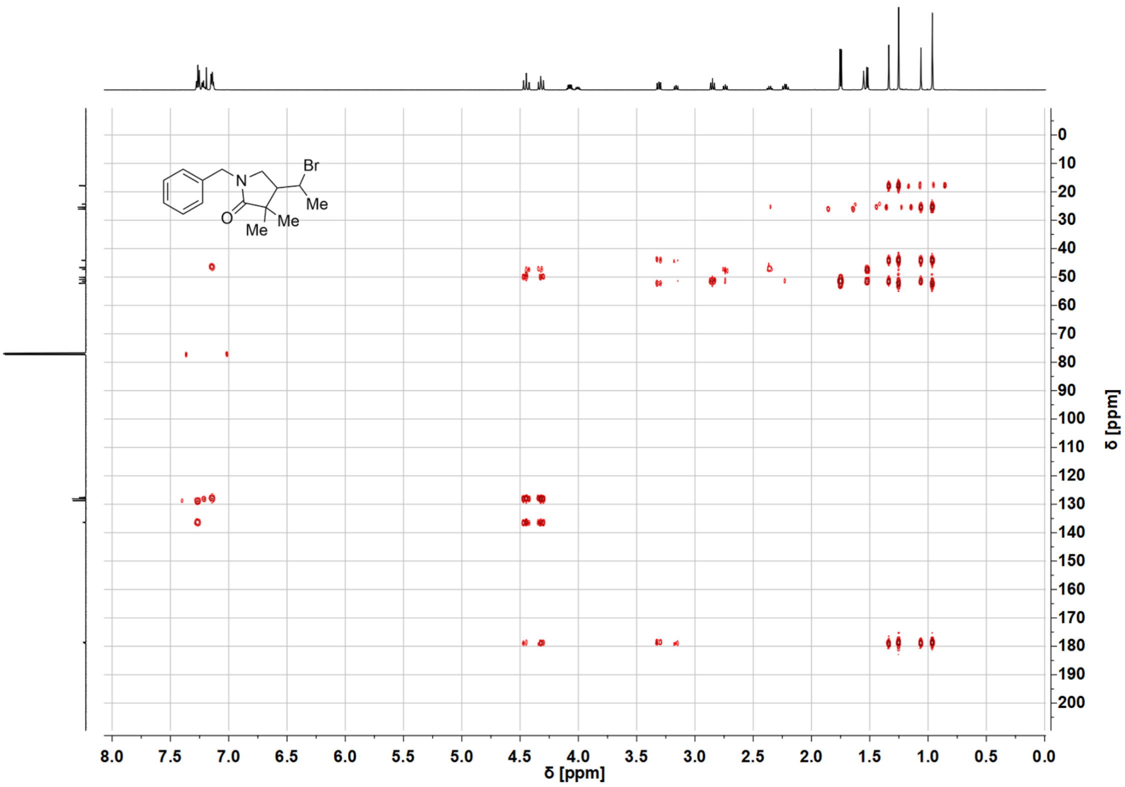

Supplement: Supplementary file 1 — Supplementary [file ANIE-59-16357-s001.pdf]
